# SCAP contributes to embryonic angiogenesis by negatively regulating KISS-1 expression in mice

**DOI:** 10.1038/s41419-023-05754-8

**Published:** 2023-04-06

**Authors:** Guo Zheng, Yu Su, Li Wei, Yingcheng Yao, Yizhe Wang, Xiaoting Luo, Xing Wang, Xiong Z. Ruan, Danyang Li, Yaxi Chen

**Affiliations:** 1 Centre for Lipid Research & Key Laboratory of Molecular Biology for Infectious Diseases (Ministry of Education), Institute for Viral Hepatitis, Department of Infectious Diseases, the Second Affiliated Hospital, Chongqing Medical University, Chongqing, 400016 China; 2 John Moorhead Research Laboratory, Centre for Nephrology, University College London Medical School, Royal Free Campus, University College London, London, NW3 2PF UK

**Keywords:** Angiogenesis, Cell signalling

## Abstract

Sterol regulatory element-binding protein (SREBP) cleavage-activating protein (SCAP) is indispensable in organ development because it maintains intracellular cholesterol homeostasis. The vessel is not widely conceived of as a cholesterol-sensitive tissue, so the specific role of SCAP in angiogenesis has not been paid attention to. As an important component of the vascular mesoderm, vascular smooth muscle cells (VSMCs) are widely involved in each step of angiogenesis. Here, we report for the first time that VSMC-specific ablation of SCAP inhibits VSMC proliferation and migration, interacting with endothelial cells (ECs), and finally causes defective embryonic angiogenesis in mice. Mechanistically, we demonstrated that SCAP ablation in VSMCs leads to the upregulation of KISS-1 protein, consequently resulting in suppressed activation of the MAPK/ERK signaling pathway and downregulation of matrix metalloproteinase 9 (MMP9) and vascular endothelial-derived growth factor (VEGF) expression to prevent angiogenesis. Importantly, we found that SCAP promotes the cleavage and nuclear translocation of SREBP2, which acts as a negative transcription regulator, regulating KISS-1 expression. Our findings suggest that SCAP contributes to embryonic angiogenesis by negatively regulating KISS-1 expression in mice and provide a new point of view for therapeutic targets of vascular development.

## Introduction

The growth and maturation of blood vessels are highly controlled multistep processes, collectively termed angiogenesis. Abnormal angiogenesis can lead to a variety of disorders, such as incomplete vascularization of the yolk sac, umbilical cord-placenta junction disorder, fetal edema, and fetal death [1–3]. The development of the embryonic vascular system is a complex process in which a variety of cells are involved and regulated by multiple signaling pathways. The well-recognized pattern of angiogenesis during vascular development is that endothelial cells (ECs) accumulate in the dorsal aorta or main vein to form a neovascularization network, recruit vascular smooth muscle cells (VSMCs) to encompass the mature vessels and undergo a complicated remodeling process to stabilize the mature vessels [4, 5]. As an important component of the vascular mesoderm, VSMC proliferation, differentiation, migration and interaction with ECs are involved in angiogenesis [6, 7]. During early development, VSMCs in the vascular wall dynamically regulate vessel diameter to supply the increasing developmental demands of the embryo [8, 9]. Deficiencies in any of the above link result in failure of angiogenesis. However, the specific mechanisms mediating the involvement of VSMCs in embryonic angiogenesis have not been fully investigated.

Sterol regulatory element-binding protein (SREBP) cleavage-activating protein (SCAP) is an endoplasmic reticulum protein that senses changes in cholesterol levels in cells. When the sterols in cells are exhausted, SCAP binds tightly to SREBPs to form a complex that is rapidly transported from the endoplasmic reticulum (ER) to the Golgi, where SREBPs are cleaved and trafficked to the nucleus to activate the transcription of genes involved in de novo cholesterol biosynthesis [10–13]. Accumulated studies have found that SCAP is indispensable in the development of important cholesterol metabolic organs or tissues, such as the brain and intestine, by maintaining intracellular cholesterol homeostasis [14–16]. Because blood vessels are not widely conceived of as cholesterol-sensitive tissues, the role of SCAP in angiogenesis currently receives insufficient attention. In a series of our previous studies, we demonstrated that SCAP in VSMCs could control and maintain vascular cholesterol homeostasis and then determine atheroma progression by regulating foam cell formation and mediating VSMC-EC communication [17, 18]. Interestingly, we recently found that SCAP ablation in VSMCs causes abnormal vascular development in the mouse placental vagus layer [19]. This study supports that SCAP contributes to angiogenesis, but there is no more direct evidence thus far.

KISS-1, an antimetastatic gene first identified in melanoma [20–22], acts biologically by binding to G protein-coupled receptor, also known as KISS-1 receptor (KISS-1R) [23, 24]. KISS-1 is involved in the gonadal system and organ development, maturation and miscarriage [25–28]. A recent study found that exogenous addition of KISS-1 inhibits vascular endothelial-derived growth factor (VEGF) expression, thereby controlling embryonic blood vessel formation and the growth of new vessels among existing vessels [29]. Kisspeptin-10, a short peptide produced from KISS-1, has also been confirmed to have angiogenic inhibitory and vasoconstrictor effects in vitro [30]. However, in vivo, the sources and functions of KISS-1 during angiogenesis are unknown. There is evidence that VSMCs can express secreted KISS-1, but the specific function of KISS-1 in VSMCs remains to be demonstrated, warranting continued research [31].

In this study, we established a model of VSMC-specific SCAP ablation in mice to investigate the role of SCAP in vascular development and angiogenesis. We found that vascular development was impaired in VSMC-specific SCAP ablation mice, manifested by thinning of the vascular wall, a decreased number of vascular cell layers, and abnormal proliferation and migration of VSMCs. All these abnormalities are related to increased KISS-1 secretion in VSMCs. Furthermore, we found that SREBP2 could bind to the KISS-1 promoter and inhibit KISS-1 transcription, thereby activating KISS-1 downstream signaling pathways to affect angiogenesis. These interesting findings suggest that SCAP performs a novel function in angiogenesis and provide a new point of view for therapeutic targets of vascular development.

## Results

### Ablation of SCAP in VSMCs leads to vessel wall thinness in mouse embryos

**Fig. 1:**
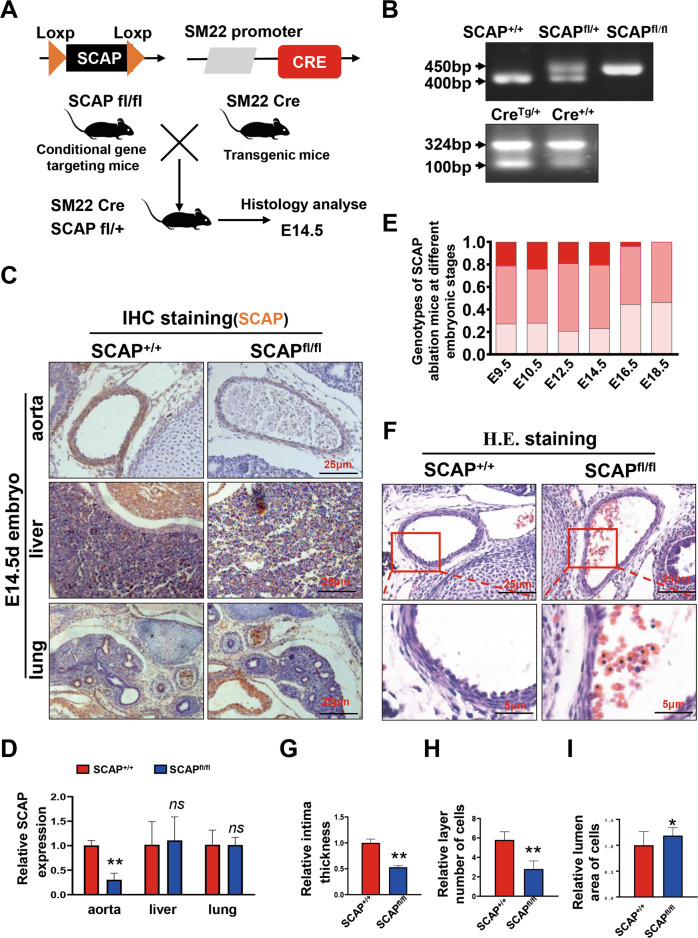
Ablation of SCAP in VSMCs leads to vessel wall thinness in mouse embryos. **A** Brief overview of the animal model generation procedure. VSMC-specific SCAP ablation mice were generated by crossing conditional gene targeting mice with conditional loxP-flanked SCAP (SCAP^flox/flox^) alleles and SM22-Cre mice. Embryos and placentas were collected on E14.5d. **B** PCR results of genotyping of mouse tails or embryonic tissue samples confirmed the generation of SM22α-Cre^+^ SCAP^flox/+^ mice, SM22α-Cre^+^ SCAP^flox/flox^ mice (SCAP^fl/fl^) and SM22α-Cre^+^ SCAP^+/+^(SCAP^+/+^). **C** Immunohistochemistry analysis of SCAP in embryonic aorta, liver, and lung (*n* = 6). Scale bar = 25 μm. **D** Statistical analysis of relative SCAP expression measured in (**C**) (*n* = 6). **E** Statistical analysis of the genotypes of SCAP ablation mice at different embryonic stages (*n* = 132). **F** H&E staining of embryonic aorta sections at E14.5 (*n* = 6). Scale bar = 25 μm. Enlarged views of the red boxes are shown. Scale bar = 5 μm. **G** Statistical analysis of the relative intima thickness in SCAP^+/+^ and SCAP^fl/fl^ mice at E14.5d (*n* = 6). **H** Statistical analysis of relative layer number of cells in SCAP^+/+^ and SCAP^fl/fl^ mice at E14.5d (*n* = 6). **I** Statistical analysis of relative lumen area of cells in SCAP^+/+^ and SCAP^fl/fl^ mice at E14.5d (*n* = 6). Data are presented as the mean ± SD. *P < 0.05, **P < 0.01, ***P < 0.001. *P* values were calculated by Student’s *t* test.

Attempts were made to generate VSMC-specific SCAP ablation mice by crossing SCAP^fl/fl^ mice with SM22-Cre transgenic mice. Since the cross silenced the expression of the first exon of SCAP, the expression of SCAP was disordered, which ultimately led its ablation (Fig. 1A). Typically, heterozygous mice were crossed to obtain homozygotes. All progeny were genotyped and verified by PCR (Fig. 1B). Immunohistochemical analysis demonstrated that SCAP expression levels were significantly lower in the aortas of homozygous mice (SCAP^fl/fl^) than in those of wild-type (SCAP^+/+^) mice (Fig. 1C, D). Genotyping of timed gestational embryos showed that self-generated SCAP^+/–^ mice from E9.5d to E14.5d exhibited the expected 25% Mendelian ratio. At E16.5d, the number of SCAP^fl/fl^ homozygous embryos was significantly reduced, and no viable homozygous mice were detected at E18.5d (Fig. 1E). This observation alerted us of homozygous embryo SCAP^fl/fl^ death during embryonic development. To gain further insight into the development of homozygous (SCAP^fl/fl^), we dissected the embryos of pregnant mice at E14.5d. Further pathological examination revealed a significant thinning of the vascular wall, a decrease in the number of cell layers and relative increase in lumen area in the canal walls of SCAP-ablated aortas (Fig. 1F–I). Considering the lethal phenotype of homozygous (SCAP^fl/fl^) embryos, these data suggested that ablation of SCAP in embryonic vessels has impacts on vascular development.

### Ablation of SCAP in VSMCs impairs VSMC proliferation in mouse embryos

**Fig. 2:**
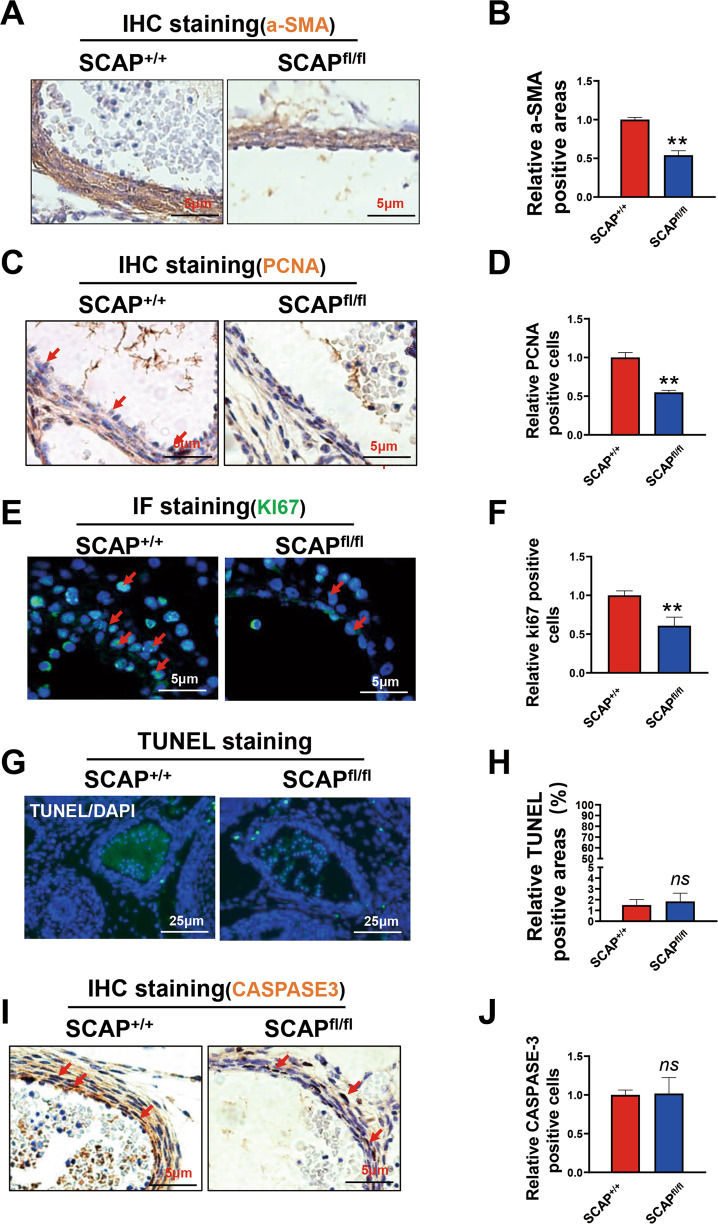
Ablation of SCAP in VSMCs impairs VSMC proliferation in mouse embryos. **A** Immunohistochemistry analysis of α-SMA in the embryonic aorta at E14.5 (*n* = 6). Scale bar = 5 μm. **B** Statistical analysis of relative α-SMA-positive areas (*n* = 6). **C** Immunohistochemistry analysis of PCNA in embryonic aortas at E14.5 (*n* = 6). Scale bar = 5 μm. **D** Statistical analysis of relative PCNA-positive cells (*n* = 6). **E** Immunofluorescence staining of Ki67 in the embryonic aorta at E14.5 (*n* = 6). Scale bar = 5 μm. **F** Statistical analysis of relative Ki67-positive cells (*n* = 6). **G** Representative images of TUNEL staining of embryonic aortas at E14.5 in each group (*n* = 6). Scale bar = 25 μm. **H** Quantification of relative TUNEL-positive areas in each group (*n* = 6). **I** Immunofluorescence staining of CASPASE-3 in the embryonic aorta at E14.5 (*n* = 6). Scale bar = 5 μm. **J** Statistical analysis of the relative number of CASPASE-3 positive cells (*n* = 6). Data are presented as the mean ± SD. *P < 0.05, **P < 0.01, ***P < 0.001. *P* values were calculated by Student’s *t* test.

To further explore the effect of SCAP ablation on embryonic vascular development, we first examined the expression of α-SMA, a marker of VSMC differentiation and maturation, which was significantly lower in homozygous embryos than in wild-type embryos (Fig. 2A, B). Next, we examined vascular proliferation and apoptosis by PCNA, KI67, TUNEL, and CASPASE-3 staining assays. PCNA and KI67 stainings showed that homozygous embryos at E14.5d had significantly reduced proliferation in aortic tissue compared with WT (Fig. 2C–F). However, TUNEL and CASPASE-3 staining results showed that apoptosis of the aortic vascular walls in the homozygous embryos was not significantly increased (Fig. 2G–J). These findings indicate that the thinner vessel walls and reduced number of vascular layers in SCAP-ablated fetuses are mainly due to impaired VSMC proliferation.

### Ablation of SCAP in VSMCs decreases VSMC proliferation and migration abilities in vitro

**Fig. 3:**
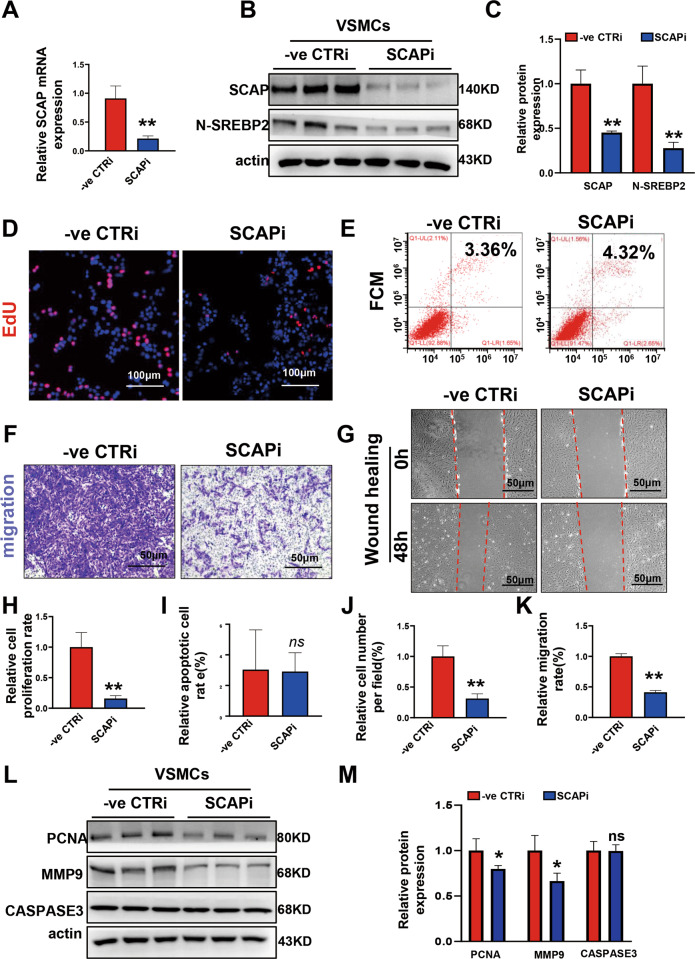
Ablation of SCAP in VSMCs decreases VSMC proliferation and migration abilities in vitro. **A** Relative mRNA expression of SCAP in VSMCs transiently transfected with SCAPi or negative control small interfering RNA (-ve CTRi). **B** Immunoblot analysis of SCAP and N-SREBP2 protein expression in CTRi and SCAPi cells. **C** Quantification of the relative protein expression of (**B**). **D** Representative images of the EdU assay. Scale bar = 50 μm. **E** Flow cytometry analysis of apoptosis in CTRi and SCAPi cells. **F** Representative images of the Transwell migration assay for each group. Scale bar = 50 μm. **G** Representative images of wound-healing migration assays at 0 h and 48 h for each group. Scale bar = 50 μm. **H** Statistical analysis of the relative cell proliferation rate in (**D**). **I** Statistical analysis of the relative apoptotic cell rate in (**E**). **J** Statistical analysis of the relative VSMC migration rate in (**F**). **K** Statistical analysis of the relative cell number per field in (**G**). **L** Immunoblot analysis of PCNA, MMP9 and CASPASE3 protein expression levels in CTRi and SCAPi cells. **M** Quantification of the relative protein expression levels in (**L**). All experiments were repeated at least 3 times. Data are presented as the mean ± SD. *P < 0.05, **P < 0.01, ***P < 0.001. *P* values were calculated by Student’s *t* test.

Next, VSMC SCAPi cell lines were established with siRNA to validate the data from in vivo experiments. RT‒PCR and Western blotting analyses showed significant interference efficiency (Fig. 3A–C). The EdU assay showed results similar to those in vivo, and cell proliferation was impaired after SCAP interference (Fig. 3D, H). Flow cytometry showed no significant increase in apoptosis after SCAP interference, which was alike to in vivo detection (Fig. 3E, I). The effect of SCAP on cell migration, a process critical to vascular development, was assessed using in vitro wound healing assays and Transwell experiments. After inducing wounds by scratching for 48 h, control cells showed more wound closure than interfering cells (Fig. 3G, K). Transwell experiments showed that inhibition of SCAP expression suppressed cell migration (Fig. 3F, J). Finally, immunoblot analysis of the protein expression levels of PCNA, matrix metalloproteinase 9 (MMP9, Important components of extracellular matrix), and CASPASE-3 in the interfering cells showed significant decreases in PCNA and MMP9 (Fig. 3L, M). These data suggest that, similar to the in vivo results, SCAP interference reduces cell proliferation and migration.

### Ablation of SCAP in VSMCs increases the expression of KISS-1 in mouse embryos

**Fig. 4:**
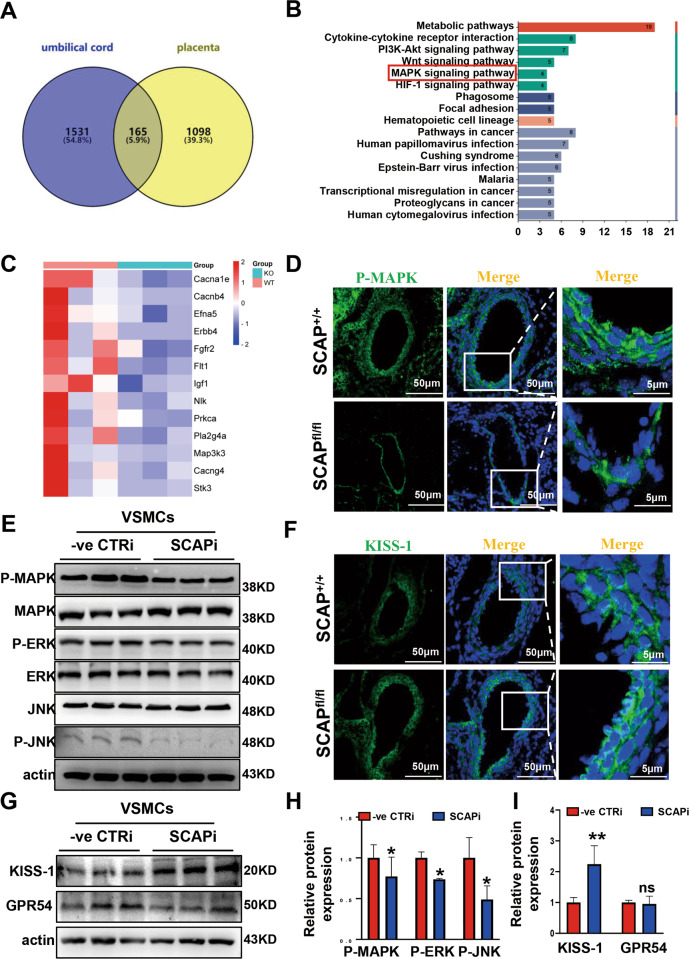
Ablation of SCAP in VSMCs increases the expression of KISS-1 in mouse embryos. **A** Venn diagram of the differentially expressed genes (DEGs) in the umbilical cord and placenta from SCAP^+/+^ and SCAP^fl/fl^ mice. **B** KEGG enrichment analysis of the 165 DEGs screened from (**A**). **C** Heatmap of the related molecules in the MAPK signaling pathway from SCAP^+/+^ and SCAP^fl/fl^ mice. **D** Immunofluorescence staining of P-MAPK in the embryonic aorta at E14.5 (*n* = 6). Scale bar = 50 μm. Enlarged merge views of the red boxes are shown. Scale bar = 5 μm. **E** Western blot analysis and quantification of MAPK, P-MAPK, ERK, P-ERK, JNK and P-JNK protein expression in CTRi and SCAPi VSMCs. **F** Immunofluorescence staining of KISS-1 in the embryonic aorta at E14.5 (*n* = 6). Scale bar = 50 μm. Enlarged merge views of the red boxes are shown. Scale bar = 5 μm. **G** Immunoblot analysis of KISS-1 and GPR54 protein expression in CTRi and SCAPi VSMCs. **H** Statistical analysis of the relative protein expression levels in (**E**). **I** Statistical analysis of the relative protein expression levels in (**G**). Data are presented as the mean ± SD. *P < 0.05, **P < 0.01, ***P < 0.001. *P* values were calculated by Student’s *t* test.

To explore the specific causes of vascular development defects in embryos, we collected mouse embryonic umbilical cord and placental tissues and analyzed and compared the transcriptional profiles of the placenta and umbilical cord using RNA-seq. The placenta data are available from the Sequence Read Archive (SRA) database (registry number SRP270041), while the umbilical cord data are not publicly available. Based on FDR-adjusted *P* < 0.05, the ablation group had 1697 differentially expressed genes (DEGs) in the umbilical cord and 1264 DEGs in the placenta compared to the WT group, and the same 165 differentially expressed genes in the umbilical cord and placenta were screened by Venn analysis (Fig. 4A). KEGG enrichment analysis was performed on the 165 DEGs, which were mainly enriched in metabolic, PI3K-Akt, MAPK/ERK, and cytokine‒cytokine receptor interaction pathways (Fig. 4B). The MAPK/ERK signaling pathway is closely related to cell proliferation and migration. Further screening of MAPK/ERK-related signaling molecules in the umbilical cord transcriptome data showed that MAPK/ERK-related signaling molecules were significantly downregulated in the ablation group (Fig. 4C). P-MAPK/P38 expression was significantly reduced in pure zygotic embryos compared to WT embryos (Fig. 4D). Similar to the results of the in vivo experiments, P-MAPK/P38, P-ERK1/2 and P-JNK protein expression levels were reduced after SCAP interference in the in vitro experiments (Fig. 4E, H). The MAPK/ERK signaling pathway is reported to be regulated intracellularly by KISS-1, and KISS-1 is associated with abortion, cell proliferation and migration. VSMCs can secrete KISS-1, but the exact mechanism is unclear [21, 28]. Then, we tested the expression level of KISS-1 in embryo vessels and found that it was more highly expressed in homozygous vessels than in WT vessels (Fig. 4F). Immunoblot analysis showed that KISS-1 protein expression was significantly elevated after SCAP interference, while the expression of the KISS-1 receptor GPR54 was not significantly changed (Fig. 4G, I). These data suggest that SCAP ablation increases KISS-1 expression and inhibits the MAPK/ERK signaling pathway, thereby affecting the proliferation and migration of VSMCs.

### Knockdown of KISS-1 expression rescues SCAP-ablated VSMCs from loss of proliferation and migration abilities in vitro

**Fig. 5:**
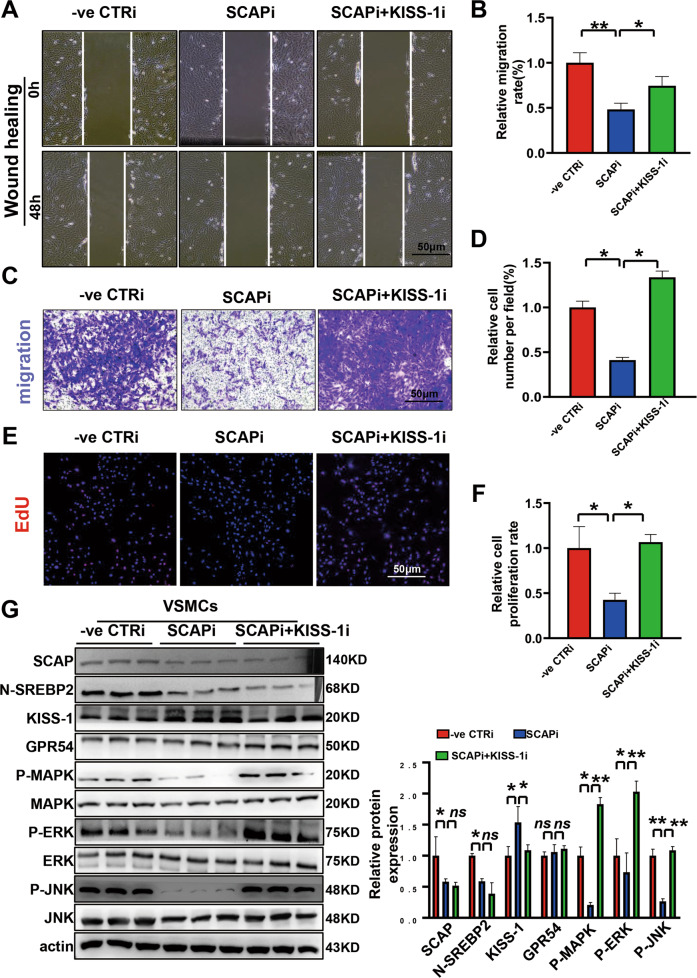
Knockdown of KISS-1 expression rescues SCAP-ablated VSMCs from loss of proliferation and migration abilities in vitro. **A** Representative images of wound-healing migration assays in VSMCs treated with CTRi, SCAPi, SCAPi and KISS-1i for 0 h and 48 h. **B** Statistical analysis of the relative VSMC migration rates in (**A**). **C** Representative images of the Transwell migration assay in VSMCs treated with CTRi, SCAPi, SCAPi and KISS-1i. **D** Statistical analysis of the relative cell numbers per field (%) in (**C**). **E** Representative images of the EdU assay in VSMCs treated with CTRi, SCAPi, SCAPi and KISS-1i. **F** Statistical analysis of the relative cell proliferation rate in (**E**). **G** Immunoblot analysis and quantification of SCAP, N-SREBP2, KISS-1, GPR54, P-MAPK, MAPK, P-ERK, ERK, P-JNK and JNK protein expression in VSMCs treated with CTRi, SCAPi, SCAPi and KISS-1i. All experiments were repeated at least 3 times. Data are presented as the mean ± SD. *P < 0.05, **P < 0.01, ***P < 0.001. *P* values were calculated by Student’s *t* test.

To determine whether SCAP is involved in vascular development through the KISS-1/MAPK/ERK signaling pathway, we used KISS-1 siRNA. Wound healing experiment showed that KISS-1 interference increased the wound closure area, while SCAP interference decreased the migration area compared to the control group (Fig. S1A, B). However, simultaneous interference of SCAP and KISS-1 significantly increased the area of wound closure compared to the SCAPi group (Fig. 5A, B). The data showed that interference with KISS-1 expression partially rescued the impaired migratory ability of cells after SCAPi. In a Transwell assay, the migration ability of cells damaged after interference with SCAP was restored by the addition of KISS-1 siRNA (Figs. 5C, D, S1C, D). The number of EdU-positive cells also increased after the addition of KISS-1 siRNA (Figs. 5E–F, S1E–F). Immunoblotting detected the protein expression levels of SCAP, N-SREBP2, KISS-1, P-MAPK/P38, P-ERK1/2 and P-JNK, and the addition of KISS-1 siRNA after SCAP interference significantly increased the expression levels of P-MAPK/P38, P-ERK1/2 and P-JNK compared with those in the SCAP interference group, indicating that reducing the expression of KISS-1 activated the expression of the MAPK/ERK signaling pathway, which was repressed after SCAP interference (Figs. 5G, S1G). These data further indicated that SCAP interference impaired cell proliferation and migration but restores the damaged ability of cells by interfering with KISS-1 expression and activating the MAPK/ERK signaling pathway.

### Ablation of SCAP in VSMCs inhibits EC function through intercellular communication

**Fig. 6:**
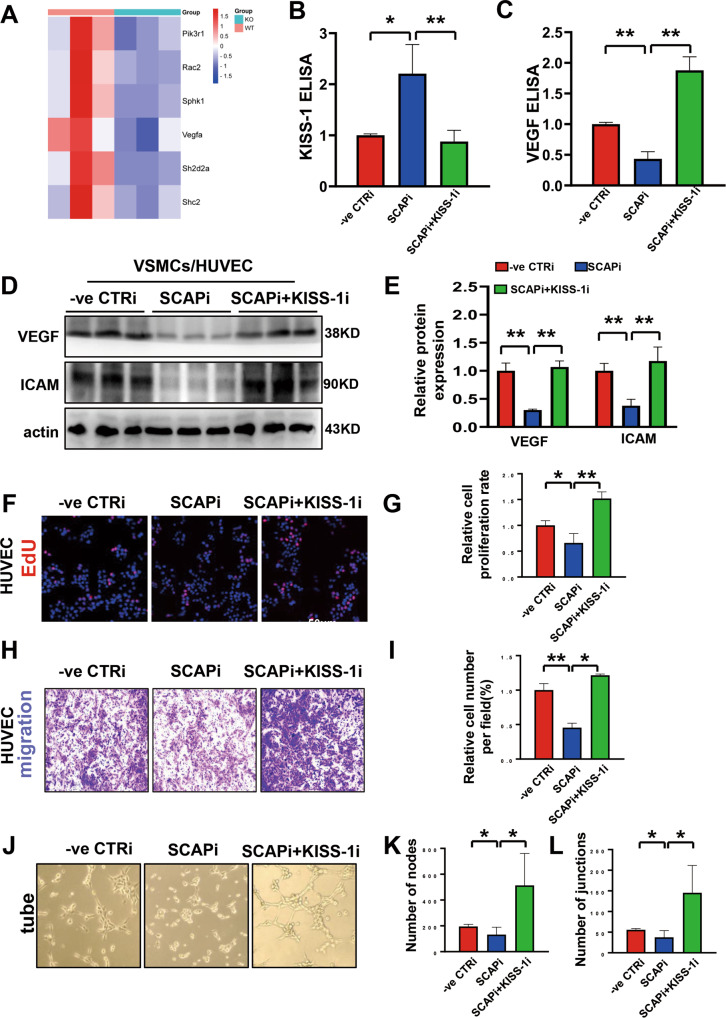
Ablation of SCAP in VSMCs inhibits endothelial cell function through intercellular communication. **A** Heatmap of the related molecules in the VEGF signaling pathway from the placentas of SCAP^+/+^ and SCAP^fl/fl^ mice. **B** Secretion concentrations of KISS-1 in culture supernatant as measured by ELISA in VSMCs treated with CTRi, SCAPi, SCAPi and KISS-1i. **C** Concentrations of VEGF in the culture supernatant as measured by ELISA in VSMCs treated with CTRi, SCAPi, SCAPi and KISS-1i. **D** Immunoblot analysis of VEGF and ICAM protein expression in HUVECs cocultured with VSMCs of different groups. **E** Statistical analysis of the relative protein expression levels in (**C**). **F** Representative images of the EdU assay of HUVECs co-cultured with supernatant from VSMCs treated with CTRi, SCAPi, SCAPi and KISS-1i. **G** Statistical analysis of the relative cell proliferation rate in (**F**). **H** Representative images of the Transwell migration assay of HUVECs co-cultured with supernatant from VSMCs treated with CTRi, SCAPi, SCAPi and KISS-1i. **I** Statistical analysis of the relative cell numbers per field (%) in (**C**). **J** Representative images of tube formation assays of HUVECs co-cultured with supernatant from VSMCs treated with CTRi, SCAPi, SCAPi and KISS-1i. **K** Quantitative analysis of number of nodes in (**J**). **L** Quantitative analysis of number of Junctions in (**J**). All experiments were repeated at least 3 times. Data are presented as the mean ± SD. *P < 0.05, **P < 0.01, ***P < 0.001. *P* values were calculated by Student’s *t* test.

KISS-1 is a secreted protein that has been shown to modulate endothelial VEGF and influence its angiogenesis [32]. In this study, we further investigated the effect of VSMC-derived KISS-1 on endothelial angiogenesis through intercellular communication. We observed that the expression of the VEGF signaling pathway in the placenta was significantly downregulated in the ablation group (Fig. 6A). The secretion of KISS-1 was increased after SCAP interference and was significantly reduced after the addition of KISS-1 siRNA (Fig. 6B). The secretion of VEGF, one of the most potent proangiogenic factors, was significantly reduced after SCAP interference and rebounded after the addition of KISS-1 siRNA (Fig. 6C). ICAM-1, an endothelial adhesion factor involved in angiogenesis, is most strongly expressed in proliferating vessels. Indirect co-culture experiment of VSMC-derived supernatant with ECs was performed to study the effect of SCAP interference on endothelial cells (Fig. S2A), and it was found that the expression levels of VEGF and ICAM-1 were significantly reduced in ECs after interference with VSMC SCAP, while the protein expression levels were elevated in endothelial cells after the addition of KISS siRNA (Fig. 6D–E). The changes of proliferation, migration and tube formation ability of endothelial cells were further detected after coculture. And the number of EdU-positive cells increased significantly (Fig. 6F–G). Transwell experiment also showed that the invasion ability was restored after KISS-1 interference (Fig. 6H–I), and tube formation ability was also increased significantly (Fig. 6J–K). Previously, it was found that VSMC SCAP interference induces angiogenesis defects in placental labyrinths [19]. In contrast to the WT placenta, the vascular branches on the surface of the homozygous placenta were disorganized and almost invisible (Fig. S2B). Immunofluorescence analysis clarified that homozygous mice had poorly formed placental vasculature (Fig. S2C, F). KISS-1 expression was significantly upregulated in the placentas of the ablation group (Fig. S2D, E). Ablation of SCAP in VSMCs promotes secretion of KISS-1 to affect endothelial cell angiogenesis, which in turn affects placental fetal angiogenesis.

### SREBP2 represses KISS-1 expression by binding to the KISS-1 promoter

**Fig. 7:**
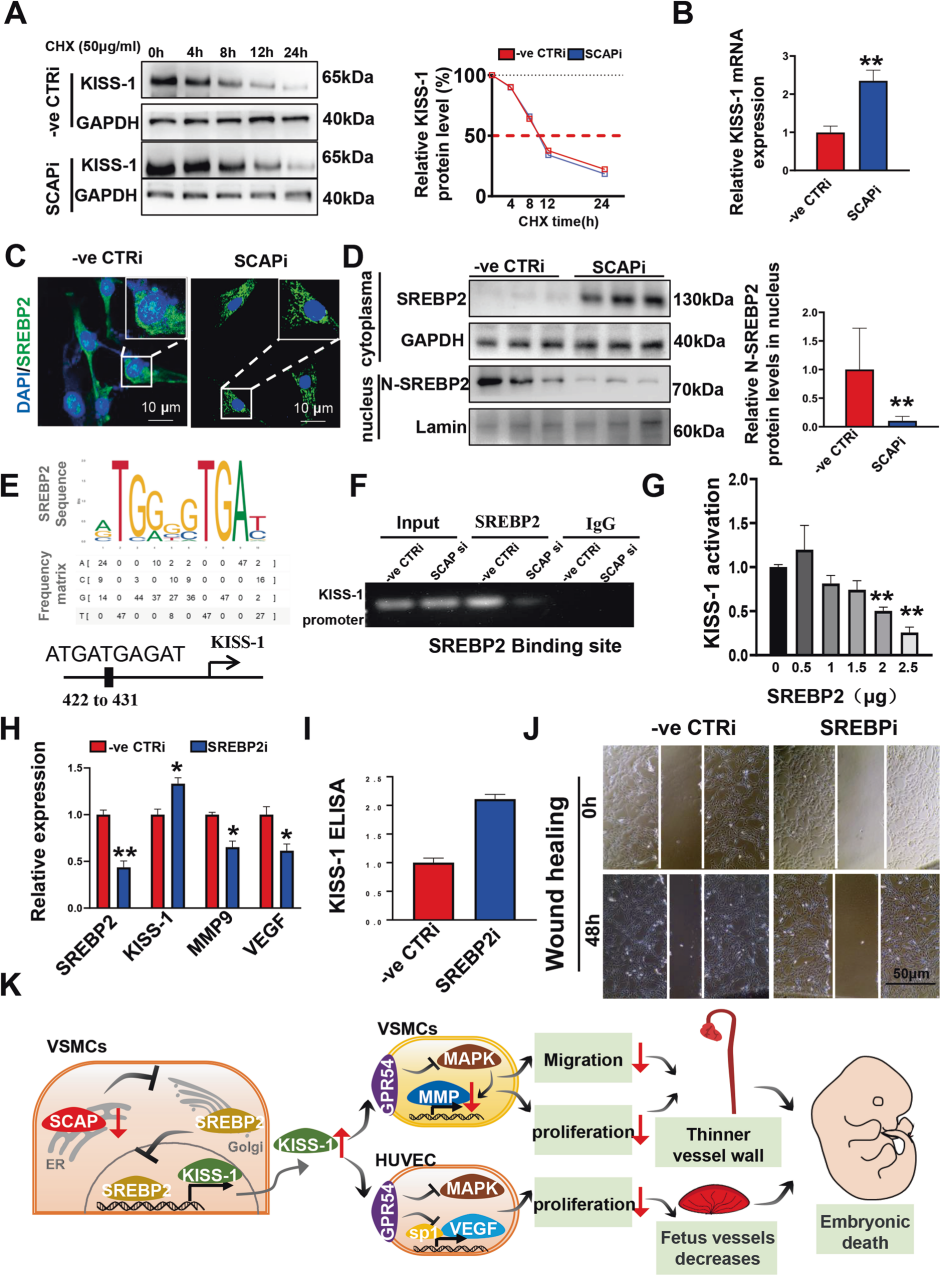
SREBP2 represses KISS-1 expression by binding to the KISS-1 promoter. **A** Half-lives of KISS-1 protein expression in CTRi and SCAPi VSMCs after treatment with 50 μg/ml cycloheximide (CHX) and harvesting at different time points as indicated. **B** Relative KISS-1 mRNA expression levels in CTRi and SCAPi VSMCs as measured by qRT‒PCR. **C** Immunofluorescence staining of SREBP2 in CTRi and SCAPi VSMCs. Scale bar = 10 μm. **D** Immunoblot analysis and quantification of SREBP2 protein expression in the nucleus and cytoplasm of CTRi and SCAPi VSMCs. **E** A putative SREBP2 binding site was predicted in the promoter region of KISS-1 (422 to 431) by the JASPAR database. **F** Agarose electrophoresis following ChIP to determine the SREBP2 binding site in the KISS-1 promoter. **G** The relative luciferase activities of KISS-1 were detected after cotransfection with the pGL3-basic luciferase reporter plasmid vector and different contents of the constructed SREBP2 plasmid for 48 h. **H** Relative mRNA expression levels of SREBP2, KISS-1, MMP9 and VEGF in CTRi and SREBPi VSMCs as measured by qRT‒PCR. **I** Secretion concentrations of KISS-1 in culture supernatant as measured by ELISA in VSMCs treated with CTRi and SREBP2i. **J** Representative images of wound-healing migration assays in VSMCs treated with CTRi and SREBP2i for 0 h and 48 h. **K** Schematic representation of how SCAP in VSMCs affects embryonic angiogenesis through negative regulation of KISS-1. All experiments were repeated at least 3 times. Data are presented as the mean ± SD. *P < 0.05, **P < 0.01, ***P < 0.001. *P* values were calculated by Student’s *t* test.

The next step was to specifically explore how SCAP affects the expression of KISS-1. The half-life of KISS-1 was first examined, and CHX experiments showed that the half-life of KISS-1 protein was not significantly altered after SCAP interference (Fig. 7A). This suggests that SCAP interference does not increase KISS-1 expression by prolonging the half-life of KISS-1, and in turn, we focused on changes in the transcriptional level of KISS-1. KISS-1 mRNA expression was significantly increased after SCAP interference, confirming that the high expression of KISS-1 was the result of increased transcriptional levels (Fig. 7B). SREBP2 is an accompanying protein of SCAP and acts as a nuclear transcription factor that can be involved in regulating the transcription of a variety of proteins [33]. After SCAP interference, SREBP2 entry into the nucleus was reduced, as shown by immunofluorescence analysis (Fig. 7C). Furthermore, we isolated and extracted proteins from the nucleus and cytoplasm, and immunoblotting more clearly showed a significant reduction in SREBP2 entry into the nucleus after SCAP interference (Fig. 7D). We speculated that SREBP2 could be involved in the transcriptional expression of KISS-1, and the prediction showed that the promoter of KISS-1 has a region (422–431) that can bind to SREBP2 (Fig. 7E). Further Ch-IP experiments confirmed that SREBP2 binds to the promoter of KISS-1 (Fig. 7F). We found that the transcriptional activity of KISS-1 gradually decreased with increasing SREBP2 content using a dual-luciferase assay (Fig. 7G). SREBP2 can negatively regulate the transcriptional level of KISS-1. We constructed the SREBP2 interfering cell line to verify our results. The transcriptional levels of MMP9 and VEGF were reduced (Fig. 7H), while the secretion of KISS-1 was also significantly increased after SREBP2 interference (Fig. 7I). In vitro Wound healing experiments also showed that the migration ability was decreased after SREBP2 interference (Fig. 7J). Under normal conditions, after SCAP translocating to the Golgi, the entry of the transcription factor SREBP2 into the nucleus was increased; SREBP2 entering the nucleus had a repressive effect on KISS-1 transcription and reduced the levels of KISS-1 transcription and secretion. When SCAP was ablated, SREBP2 entry into the nucleus decreased, the transcriptional repression of KISS-1 was reduced, and the expression of KISS-1 increased. KISS-1 inhibits the expression of MMP-9 in VSMC through MAPK signaling pathway [34], and affects the proliferation and migration function of VSMC; KISS-1 can directly inhibit the transcription of VEGF by binding to sp1 [30], and affect the function of ECs through intercellular communication, thus affecting the process of embryonic angiogenesis (Fig. 7K).

## Discussion

Recent research has identified a number of developmental roles of SCAP in embryos, but the precise mechanism in each organ is largely unknown. As a critical molecule for maintaining intracellular cholesterol homeostasis, SCAP is indispensable for embryogenesis. Targeted disruption of SCAP in the cholesterol-rich organs of mice caused organ dysgenesis, although these models do not exhibit absolute lethality. Brains contain the highest levels of cholesterol in the body, and SCAP deletion in astrocytes showed microcephaly, without effects on astrocyte survival [16]. The intestine is commonly known to be the primary place where cholesterol is digested and absorbed, and SCAP deletion results in severe injury to intestinal epithelia and death [15]. Unlike the intestine, the liver is able to tolerate loss of SCAP, and it is perhaps relevant that SREBPs could be activated in a SCAP-independent manner and thus maintain cellular homeostasis [35, 36]. Herein, we report for the first time that VSMC-specific ablation of SCAP induces embryonic lethality due to defective blood vessel development, indicating an essential role in cardiovascular system development. This finding is interesting considering that the vasculature is not widely conceived of as a cholesterol-sensitive tissue, which warrants further investigation.

The cardiovascular system is among the earliest functional and formed organs in vertebrate embryos. Vascular development is a complex process involving many different cells and signal pathways. Initially, endothelial cells form the lumen, then recruit smooth muscle cells to form an elastic layer around the lumen and begin to construct a vascular matrix containing elastin to form multiple vascular walls. Monocyte migrasomes are rich in angiogenic factors. Purified migrasomes promote capillary formation and monocyte recruitment in vivo and endothelial tube formation and monocyte chemotaxis in vitro [37]. VSMC-derived exosomes also have angiogenic activities in vivo [38]. And the communication between various cells coordinates vascular development and maintains vascular homeostasis. VSMC proliferation, migration, recruitment and differentiation are critical processes during the development and growth of the vasculature [39, 40]. Our in vitro and in vivo data provide clear evidence that ablation of SCAP results in reductions in cell proliferation, migration, and invasion of VSMCs but does not affect apoptosis. Mechanistic studies further identified KISS-1 as one of the critical target genes of SCAP that mediates physiological angiogenesis during embryonic development. G—protein—coupled receptor (GPCR) is a kind of superfamily protein receptor. GPR54, a classic GPCR, was found to bind KISS-1 specifically in 2001 and play a key role in physiological homeostasis. Since GPR54 is a membrane protein and SCAP is a protein in the endoplasmic reticulum and Golgi apparatus, interaction between the two is highly unlikely. Therefore, we did not discuss in detail the changes of GPR54 after SCAP knockout. KISS-1 was identified as an essential regulator in hormone release and plays a critical role in physiological homeostasis. A recent body of evidence reports that KISS-1 is considered to be a potential tumor suppressor in a number of different tumors and is involved in cell proliferation, migration and invasion by targeting the MAPK/ERK and P50/MMP-9 pathways [21, 24, 34, 41]. This is in agreement with our observations. However, we also noted that our findings are inconsistent with some other studies, which have suggested that KISS-1 can also induce apoptosis in tumor cells [42–44]. This might be relevant for cell type-specific effects or may indicate that KISS-1 assumes different roles under a variety of physiological and pathological conditions.

Moreover, KISS-1 is a potent inhibitor of tumor metastasis and plays a role in placentation; both processes involve angiogenesis. The results of a previous study as well as this study show that the effects of KISS-1 were demonstrated against pathological angiogenesis in tumor models as well as against physiological angiogenesis occurring during organ development [45]. Mechanistically, KISS-1 inhibits angiogenesis by selectively blocking Sp1-dependent VEGF expression in endothelial cells, both in pathological and physiological conditions [30]. It is worth noting that the origin of KISS-1 is different in different states. KISS-1 is secreted by tumor cells in the tumor microenvironment in pathological states [46] but is secreted from placental villous trophoblasts during placental development [47], but is secreted from placental villous trophoblasts during placental development [48]. However, in arterial development, the source of KISS-1 is unknown. In humans, vasculature KISS-1 has restricted localization to the smooth muscle of vessels with the same developmental origins, umbilical vein, coronary artery and aorta [45]. Here, our data provide further evidence that vascular smooth muscle-derived KISS-1 is a key factor influencing the structural and functional development of the vasculature.

Subsequently, we tried to clarify the potential regulatory mechanism underlying the interaction between SCAP and KISS-1. The expression of KISS-1 is regulated by mutations, epigenetic and transcriptional silencing, posttranslational regulation, and protein–protein interactions [49]. Our several lines of evidence show that KISS-1 protein level is regulated by mRNA expression rather than the control of protein stability. The SCAP/SREBP complex can traffic to the Golgi, resulting in SREBP cleavage and its nuclear function. SREBP2 has been shown to be responsible for the regulation of transcription of a variety of genes, such as HMG-CoA and LDLr [50]. Moreover, there are well-known examples of SREBPs acting as transcriptional repressors, such as SREBPs repressing IRS2 transcription in the liver and repressing ABCA1 transcription in foam cells [51, 52]. Our study provides strong evidence suggesting that SREBP2 is a negative regulator of KISS-1 and thus plays an important role in angiogenesis during embryonic development.

Based on the significant value of angiogenesis in embryonic development, tissue repair, organoid cultures and tumor invasion and metastasis, our results are particularly valuable. We demonstrated that SCAP is a critical regulatory factor in angiogenesis via suppression of KISS-1. SCAP regulates KISS-1 expression through the activation of SREBP2, which acts as a transcriptional repressor to inhibit KISS-1 promoter activity, promotes the proliferation and migration of VSMCs by activating the MAPK/ERK pathway and upregulates VEGF expression via communication between VSMCs and ECs to maintain angiogenesis. Our findings provide theoretical support for further elucidating the involvement of smooth muscle-derived SCAP in angiogenesis, offer new clues for exploring vascular development, and supply new targets for the diagnosis and treatment of clinical angiogenesis-related diseases.

## Methods

### Animals

To generate VSMC-specific SCAP ablation mice, mice with conditional loxP-flanked SCAP (SCAP^loxp/loxp^) alleles were crossed with transgenic mice expressing Cre recombinase under the control of the mouse smooth muscle protein 22 promoter. Embryos and placentas were collected and analyzed on Day 14.5 of the embryonic period (E14.5). All transgenic mice were purchased from the Jackson Laboratory (Bar Harbor, ME, USA). Animals were housed under specific pathogen-free conditions. All animal studies were performed in accordance with institutional guidelines and with the National Institutes of Health Guide for the Care and Use of Laboratory Animals (National Research Council, 8th Edition, 2011) and approved by the Animal Ethics Committee of Chongqing Medical University.

### Immunohistochemical (IHC) staining

Steps were performed as described in the manufacturer’s description (sp-9001, ZSGB-BIO, Beijing, China). Paraffin-embedded sections (5 μm) were deparaffinized and hydrated, and then antigen retrieval was performed in a microwave using citric acid buffer (pH 6.0). Endogenous peroxidase activity and nonspecific antigens were blocked with peroxidase-blocking reagent and goat serum, followed by incubation with primary antibodies, including anti-SCAP (ab153933, Abcam, Cambridge, UK), anti-α-SMA (14395-1-AP, Proteintech, Wuhan, China), anti-PCNA (#13110, Cell Signaling Technology, Boston, USA), and anti-CASPASE3 (#9662, Cell Signaling Technology, Boston, USA). Then, the tissues were incubated with a goat anti-rabbit IgG/TRITC secondary antibody (ZF-0316, ZSGB-BIO, Beijing, China) and subsequently incubated with streptavidin-conjugated horseradish peroxidase. The peroxidase reaction was performed using DAB substrate (ZLI-9018, ZSGB-BIO, Beijing, China) with hematoxylin nuclear counterstaining. Slices were visualized using a Pannoramic Flash DESK DX (3DHISTECH, Budapest, Hungary). For quantification of staining, positively stained cells were counted manually using the Cell Counter function of ImageJ.

### Hematoxylin and eosin (HE) staining

Briefly, paraffin-embedded sections (5 μm) were deparaffinized, hydrated and stained in hematoxylin (C0105, Beyotime, Shanghai, China) for 2 min, washed with tap water for 10 min, and then placed in eosin (C0105, Beyotime, Shanghai, China) for 1 min, followed by dehydration and vitrification. Finally, slices were mounted with coverslips using neutral balsam (#G8590, Solarbio, Beijing, China). Tissues were visualized using a Pannoramic Flash DESK DX and analyzed by ImageJ software.

### TUNEL staining

The assay was conducted according to the manufacturer’s protocol (G3250, Promega, Madison, WI, USA). To detect DNA strand breaks occurring in apoptotic cells, frozen sections were fixed with 4% paraformaldehyde and incubated with Proteinase K solution. Next, 50 μl of rTdT incubation buffer was added to slices at 37 °C for 60 min in a dark humidified chamber. Slices were counterstained with DAPI and photographed using a Leica confocal microscope (Weztlar, Germany).

### Cell culture

Mouse vascular smooth muscle cells (VSMCs), human umbilical vein endothelial cells (HUVECs) and 293 T cells were obtained from American Type Culture Collection (ATCC, VA, USA). VSMCs were cultured in basal DMEM/F12 medium (HyClone, Logan, Utah, USA), HUVECs were grown in RPMI 1640 medium (HyClone, Logan, Utah, USA), and 293 T cells were maintained in DMEM/high-glucose medium (HyClone, Logan, Utah, USA). The complete culture medium was supplemented with 10% fetal bovine serum (HyClone, Logan, Utah, USA) and 100 U/mL penicillin‒streptomycin (Gibco, Thermo Fisher Scientific, Eugene, Oregon, USA). For coculture experiments, HUVECs were cocultured with supernatant from VSMCs treated with different siRNAs obtained in advance. All cells were maintained at 37 °C in 5% CO_2_.

### siRNA transfection

Knockdown of SCAP, KISS-1 or SREBP2 was performed by transient transfection according to the instructions for reverse small interfering RNA transfection (Tsingke, Beijing, China) in six-well plates. For each well with 30–50% confluence, 5 μl Lipofectamine RNAi MAX (13778150, Invitrogen, Waltham, UK), 20 nM mouse SCAP siRNA (SCAPi), KISS-1 siRNA (KISS-1i), SREBP2 siRNA (SREBP2i) and negative control (-veCTRi) (Tsingke, Beijing, China) were diluted with 250 μl Opti-MEM (Thermo Fisher Scientific, Mass, Waltham, USA) and then mixed together, and the transfection mixture was incubated at room temperature for 15 min. Cell suspensions in complete medium without antibiotics were incubated with the transfection mixture for 24 h. Specific siRNAs involved are as follows: mouse SCAP siRNA (SCAPi) (sense: 5ʹ-CCUCCUGGCAGUAGAUGUAdTdT-3ʹ, antisense: 5ʹ-UACAUCUACUGCCAGGAGGdTdT3ʹ), mouse KISS-1 siRNA (5ʹ-GCCGAACUACAACUGGAACTT-3ʹ). mouse SREBP2 siRNA (sense: 5ʹ- GCGGACAACACACAAUAUCAU-3ʹ, antisense: 5ʹ- GAUGCUACAGUUUGUCAGCAA 3ʹ).

### Immunoblotting

Total cellular protein was lysed in RIPA lysis buffer (P0013D, Beyotime Biotechnology, Jiangsu, China). Cytoplasmic and nuclear proteins were extracted using the NE-PER Nuclear and Cytoplasmic Extraction Kit (78833, Thermo Fisher Scientific, Waltham, Massachusetts, USA). Equal protein contents were electrophoresed using 7.5% and 12.5% dodecyl sulfate sodium dodecyl sulfate polyacrylamide gel electrophoresis (SDS‒PAGE) (PG111, PG113, YAMEI, Shanghai, China) and electrotransferred onto polyvinylidene difluoride membranes (IEVH85R, Millipore, Burlington, MA, USA). After blocking with 3% Bovine Serum Albumin(9048-46-8, Genview, Florida, USA) for 1 h at room temperature, membranes were incubated at 4 °C overnight with the following antibodies: anti-SCAP (ab153933, Abcam, Cambridge, UK), anti-SREBP2 (ab30682, Abcam, Cambridge, UK), anti-PCNA (#13110, Cell Signaling Technology, Boston, USA), anti-MMP9 (sc-393859, Santa Cruz, USA), anti-CASPASE3 (#9662, Cell Signaling Technology, Boston, USA), anti-KISS-1 (sc-18134, Santa Cruz, USA), anti-GPR54 (#13776, Cell Signaling Technology, Boston, USA), anti-MAPK/P38 (#8690, Cell Signaling Technology, Boston, USA), anti-P-P38 MAPK (Thr180/Tyr182) (#4511, Cell Signaling Technology, Boston, USA), anti-ERK (#4695, Cell Signaling Technology, Boston, USA), anti-P-P44/42 MAPK (ERK1/2) (Thr202/Tyr204)(#4370, Cell Signaling Technology, Boston, USA), anti-JNK (#9252, Cell Signaling Technology, Boston, USA), anti-P-JNK (Thr183/Tyr185)(# 4668, Cell Signaling Technology, Boston, USA), anti-VEGF (ab52917, Abcam, Cambridge, UK), anti-ICAM (10020-1-AP, Proteintech, Wuhan, China), anti-Lamin (12987-1-AP, Proteintech, Wuhan, China), anti-GAPDH (10494-1-AP, Proteintech, Wuhan, China), and anti-β-actin (20536-1-AP, Proteintech, Wuhan, China).

The primary antibody incubation was followed by incubation with HRP-conjugated corresponding secondary antibodies (SA00001-1, SA00001-2, Proteintech, Wuhan, China). Finally, the protein bands were visualized by utilizing High ECL Enhanced Western Blotting Substrate (BG0015, BIOGROUND, Chongqing, China) and photographed on an integrated chemiluminescence imaging system. The expression of the target proteins was further measured by ImageJ software with normalization to β-actin.

### Real-time quantitative PCR (RT‒qPCR)

Total RNA was extracted from cells using an RNA isolater (R401-01, Vazyme, Nanjing, China) according to a standard protocol and then reverse transcribed to cDNA with HiScript II qRT SuperMix (R222-01, Vazyme, Nanjing, China). RT‒qPCR was performed using SYBR Green PCR Master Mix (9109, Takara, Japan) with specific primers, including SCAP: forward (5ʹ-3ʹ): AAGGGACCAGGTGGAAC; reverse (5ʹ-3ʹ): GCGCGGCCACCTTGTA; KISS-1: forward (5ʹ-3ʹ): AGCCGCCAGATCCCCGC; and reverse (5ʹ-3ʹ): GCCGAAGGAGTTCCAGTTGTAGTT, SREBP2: forward (5ʹ-3ʹ): GCAGCAACGGGACCATTCT; reverse (5ʹ-3ʹ): CCCCATGACTAAGTCCTTCAACT, MMP9: forward (5ʹ-3ʹ): AGACGACATAGACGGCATCC; reverse (5ʹ-3ʹ): TGGGACACATAGTGGGAGGT, VEGF: forward (5ʹ-3ʹ): CCAGGTTTAAGCTCGGTTGCTG; reverse (5ʹ-3ʹ): GTGTGGCTTTCCACCGCCTCTC via a CFX Connect Real-time System (Bio-Rad, Hercules, CA, USA). The relative protein expression was calculated by using the 2− ΔΔCt method with the housekeeping gene β-actin as a reference gene.

### 5-Ethynyl-2′-deoxyuridine (EdU) assay

VSMCs were seeded on coverslips in a 24-well plate before SCAP siRNA transfection. Following the instructions for the BeyoClick™ EdU-555 Cell Proliferation Kit (C0075S, Beyotime, Nanjing, China), -ve CTRi VSMCs and SCAPi VSMCs were separately cocultured with 20 μM EdU working solution away from light for 2 h at 37 °C. After that, the cells were fixed in 4% paraformaldehyde and permeabilized with 0.3% Triton X-100. Next, the samples were incubated with Click Reaction Buffer and counterstained with DAPI. Fluorescence images were captured with a Leica laser scanning confocal microscope (Weztlar, Germany). The number of EdU-positive cells was quantitated by ImageJ software.

### Wound-healing assay

In brief, when VSMCs had grown to 90% confluence in six-well plates, the cell monolayer was scratched with a sterile 200 µL pipette tip and maintained in serum-free medium. After incubation for 48 h, the scratch area was imaged under a microscope (Evos XL Core, Thermo Fisher Scientific) and further measured via ImageJ software.

### Transwell migration assay

For the Transwell migration assay, -ve CTR VSMCs and SCAPi, KISS-1i or SREBP2i VSMCs were seeded in basal DMEM/F12 serum-free medium in Transwell inserts (8.0-μm pore size) (Falcon™ Cell Culture Inserts, Corning, Inc.) for 48 h, and basal DMEM/F12 medium with 10% serum was added to the lower chamber. Subsequently, crystal violet was used to stain the invaded cells through the polycarbonate membrane for 20 min. Cells were observed and counted under a microscope (Evos XL Core, Thermo Fisher Scientific, Waltham, Massachusetts, USA) in five randomly selected images per chamber.

### RNA-sequencing (RNA-seq) of umbilical cord tissues

Umbilical cord tissues from SCAP^+/+^ and SCAP^fl/fl^ mice at E14.5d were dissected and collected for RNA-sequencing analysis (Majorbio, Shanghai, China). Heatmaps were constructed and KEGG enrichment analyses of differentially expressed genes (DEGs) from transcriptomic data were performed using the DAVID and KOBAS bioinformatic resources. The raw sequence data are undisclosed online.

### Immunofluorescence (IF) staining

The frozen tissue sections or cells were allowed to recover to room temperature and then fixed with 4% PFA for 15 min, followed by permeabilization with 0.3% Triton X-100 for 15 min. Afterward, nonspecific antigen was blocked with 3% BSA for 1 h. Primary antibodies were added to the tissue and incubated at 4 °C overnight. On the following day, fluorescence-labeled secondary antibodies (Alexa Fluor 488 or tetramethylrhodamine conjugated, ZSGB-BIO, Beijing, China) were applied for 1 h at 37 °C in the dark. Finally, DAPI dye was used for nuclear counterstaining. All the primary antibodies were as follows: anti-Ki67 (28074-1-AP, Proteintech, Wuhan, China), anti-CASPASE3 (19677-1-AP, Proteintech, Wuhan, China), anti-P-MAPK (#4511, Cell Signaling Technology, Boston, USA), anti-KISS-1 (sc-18134, Santa Cruz, USA), anti-TER119 (sc-19592, Santa Cruz, USA), anti-SREBP2 (ab30682, Abcam, Cambridge, MA, UK), and anti-CD9 (#98327 S, Cell Signaling Technology, Boston, USA). All images were captured on a Leica confocal microscope (Weztlar, Germany).

### Flow cytometry

Cell apoptosis was detected using an Annexin V/PI detection kit (#559763, BD Biosciences, San Jose, USA). Briefly, CTRi and SCAPi VSMCs were harvested after transfection and washed with cold PBS. Then, 5 µl Annexin V and 5 µl 7-AAD were added and incubated for 15 min in the dark. Finally, the samples were analyzed by flow cytometry (BD Bio-sciences, US) within 1 h. Data were analyzed using FlowJo software.

### Tube formation assay

200ul Matrigel (Corning, 354234) was pre-coated in a 24-well plate and coagulated in a 37 °C incubator for 30 min. The suspension of HUVECs were added proportionally to the Matrigel and incubated with the supernatant from VSMCs treated with CTRi, SCAPi, SCAPi and KISS-1i. Visualized the formation of the tube using a microscopy (Leica, USA) after 4 h. The number of junctions and the number of nodes were quantified using ImageJ software.

### Reporting Summary

Further information on research design is available in the Nature Portfolio Reporting Summary linked to this article.

## ELISA

After specific treatment, the cell culture supernatants of each group were collected and centrifuged for 10 min at 1000 rpm. The levels of KISS-1 and VEGF in VSMC supernatants were detected using enzyme-linked immunosorbent assay (ELISA) kits purchased from the Nanjing Jiancheng Bioengineering Institute (Nanjing, China).

### Cycloheximide (CHX) chase experiment

After -ve CTR VSMCs and SCAPi VSMCs adhered to 6-cm plates, cell proteins were extracted at 0, 4, 8, 12, and 24 h with the addition of 50 μg/ml CHX (HY-12320, MedChemExpress, New Jersey, USA) and analyzed by Western blotting to investigate the half-life of KISS-1 protein.

### Chromatin immunoprecipitation (Ch-IP) assay

A Ch-IP Assay Kit (ab500, Abcam, Cambridge, MA, UK) was used according to the manufacturer’s instructions. Beads and anti-SREBP2 antibody were used to pull down the target DNA. Crosslinked DNA was sheared to a fragment size of 200-1000 base pairs using a sonicator and amplified by RT‒PCR. Finally, qPCR products were identified by agarose electrophoresis to analyze the enrichment of DNA fragments in the SREBP2 transcription binding sites in KISS-1 promoters. The results were normalized to input DNA. The following primers were used: KISS-1: Forward ((5ʹ-3ʹ): AGCCGCCAGATCCCCGC; Reverse (5ʹ-3ʹ): GCCGAAGGAGTTCCAGTTGTAGTT).

### Dual-luciferase reporter assay

The regions of the KISS-1 promoter were cloned into the pGL3-basic luciferase reporter plasmid vector (Promega, Madison, WI, USA). Later, the pGL3-basic luciferase reporter plasmid vector and different contents of the constructed SREBP2 plasmid were transiently cotransfected into 293 T cells at 70-80% confluence. After transfection for 48 h, a Dual-Lumi™ Luciferase Reporter Gene Assay Kit (RG009, Beyotime, Nanjing, China) was used to assess luciferase activity based on the manufacturer’s protocol. The luciferase activities were normalized to the activity produced by the Renilla pRLTK plasmid (Promega, Madison, WI, USA).

### Statistical analysis

All data are presented as the means ± SDs. Statistical significance was calculated by 2-tailed Student’s *t* test and one-way ANOVA using GraphPad Prism 8.4.3 software (GraphPad Software Inc., San Diego, CA, USA), with *P* < 0.05 considered significant.

## Supplementary information

supplementary figures

Supplement Figure1

Supplement Figure2

Original western blots

Reporting Summary-a reproducibility checklist

## Data Availability

Data openly available in a public repository.
