# Supplementary material for: SCAP contributes to embryonic angiogenesis by negatively regulating KISS-1 expression in mice
Source: Cell Death Dis. 2023 Apr 6;14(4):249. doi: 10.1038/s41419-023-05754-8 (PMC10079761; doi:10.1038/s41419-023-05754-8)
Supplement: Supplementary file 1 — supplementary figures [file 41419_2023_5754_MOESM1_ESM.docx]

**Legends**

**S Fig. 1.** Knockdown of KISS-1 expression promotes the proliferation and migration abilities of VSMCs in vitro**.** (A) Representative images of wound-healing migration assays in VSMCs treated with CTRi and KISS-1i for 0 h and 48 h. (B) Statistical analysis of the relative VSMC migration rate in (A). (C) Representative images of the Transwell migration assay in VSMCs treated with CTRi and KISS-1i. (D) Statistical analysis of the relative cell numbers per field (%) in (C). (E) Representative images of the EdU assay in VSMCs treated with CTRi and KISS-1i. (F) Statistical analysis of the relative cell proliferation rate in (E). (G) Immunoblot analysis and quantification of SCAP, N-SREBP2, KISS-1, GPR54, P-MAPK, MAPK, P-ERK, ERK, P-JNK and JNK protein expression in VSMCs treated with CTRi and KISS-1i. All experiments were repeated at least 3 times. Data are presented as the mean ± SD. *P < 0.05, **P < 0.01, ***P < 0.001. P values were calculated by Student’s t test.

**S Fig. 2.** Ablation of SCAP in VSMCs inhibits vascular development in vivo. (A) Schematic diagram of co-culture strategy showing the ECs co-cultured with supernatant from VSMCs. (B) Representative images of placentas of SCAP^+/+^ and SCAP^fl/fl^ mice at E14.5 (n = 6). Scale bar = 1 mm. Enlarged views photographed by stereomicroscope are shown. Scale bar = 200 μm. (C) Immunofluorescence staining of CD9 and TER119 in embryonic placentas at E14.5 (n = 6). Scale bar = 25 μm. Enlarged views are shown. Scale bar = 10 μm. (D) Immunofluorescence staining of KISS-1 in placentas at E14.5d from SCAP^+/+^ and SCAP^fl/fl^ mice (n = 6). Scale bar = 10 μm. (E) Statistical analysis of the relative KISS-1 expression levels in (D) (n = 6). (F) Statistical analysis of relative vascular density (% of control) in (C) (n = 6). Data are presented as the mean ± SD. *P < 0.05, **P < 0.01, ***P < 0.001. P values were calculated by Student’s t test.

**S FIRURES**

**
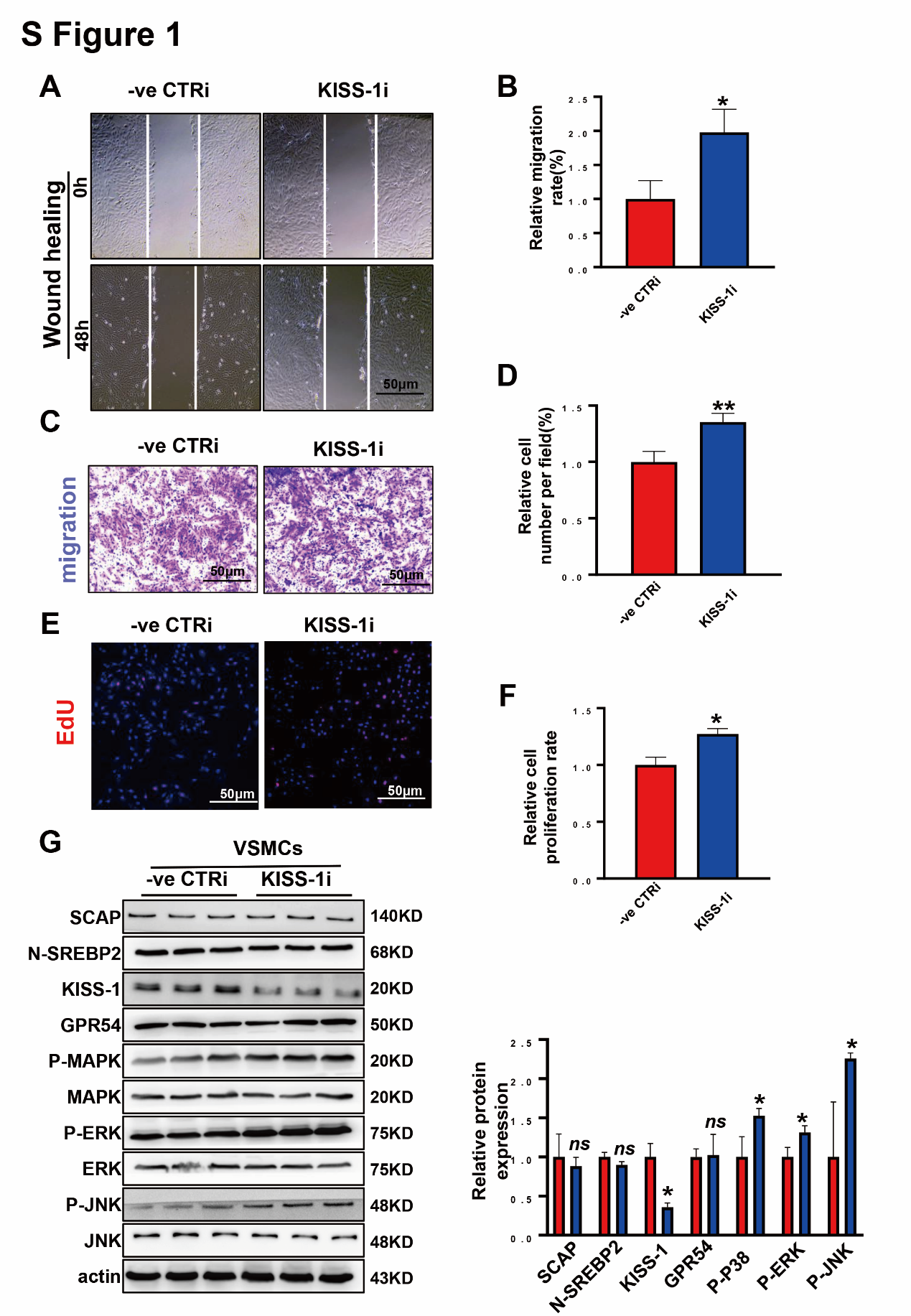
**

**
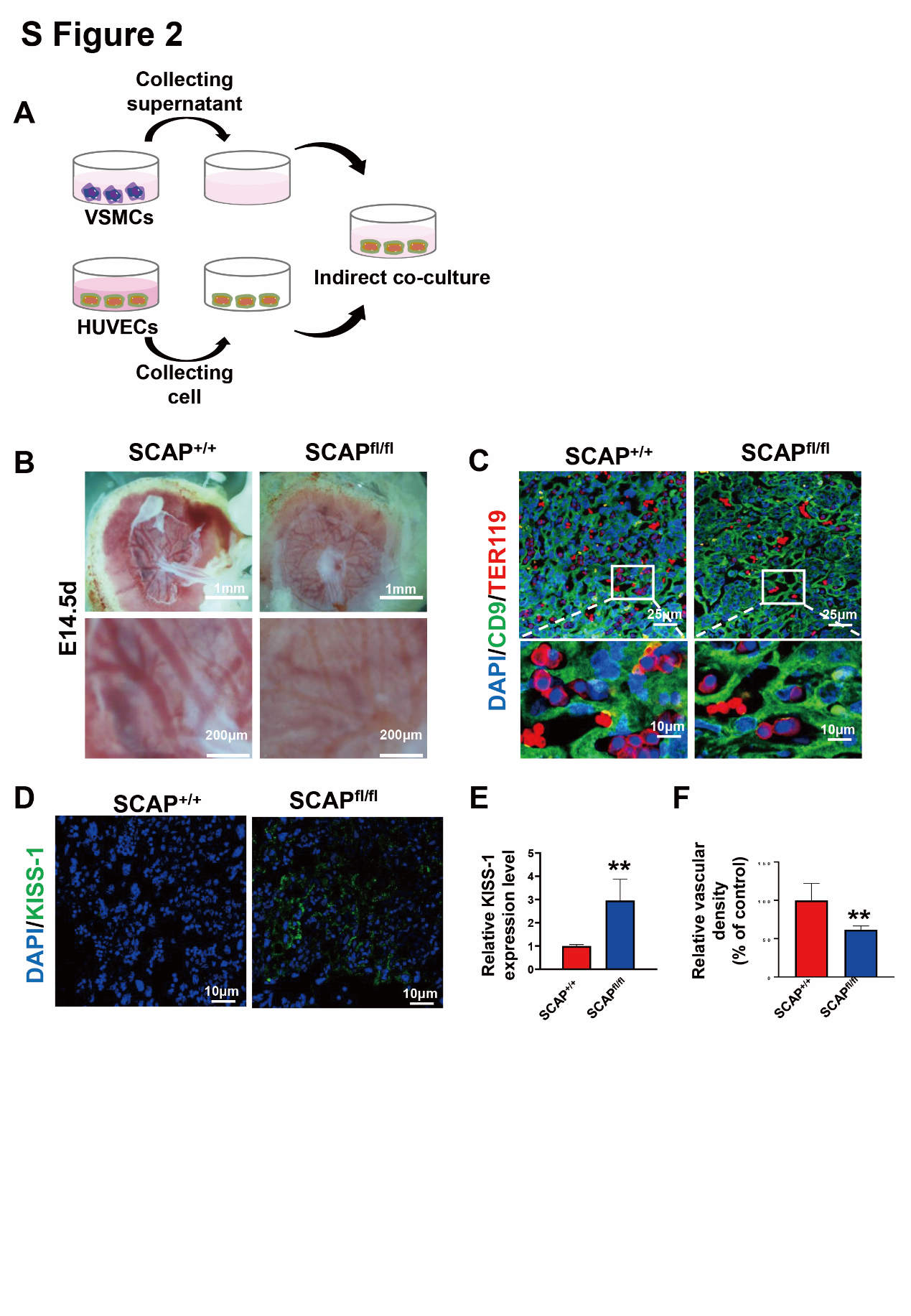
**
