# Supplementary figures and images for: SCAP contributes to embryonic angiogenesis by negatively regulating KISS-1 expression in mice
Source: Cell Death Dis. 2023 Apr 6;14(4):249. doi: 10.1038/s41419-023-05754-8 (PMC10079761; doi:10.1038/s41419-023-05754-8)

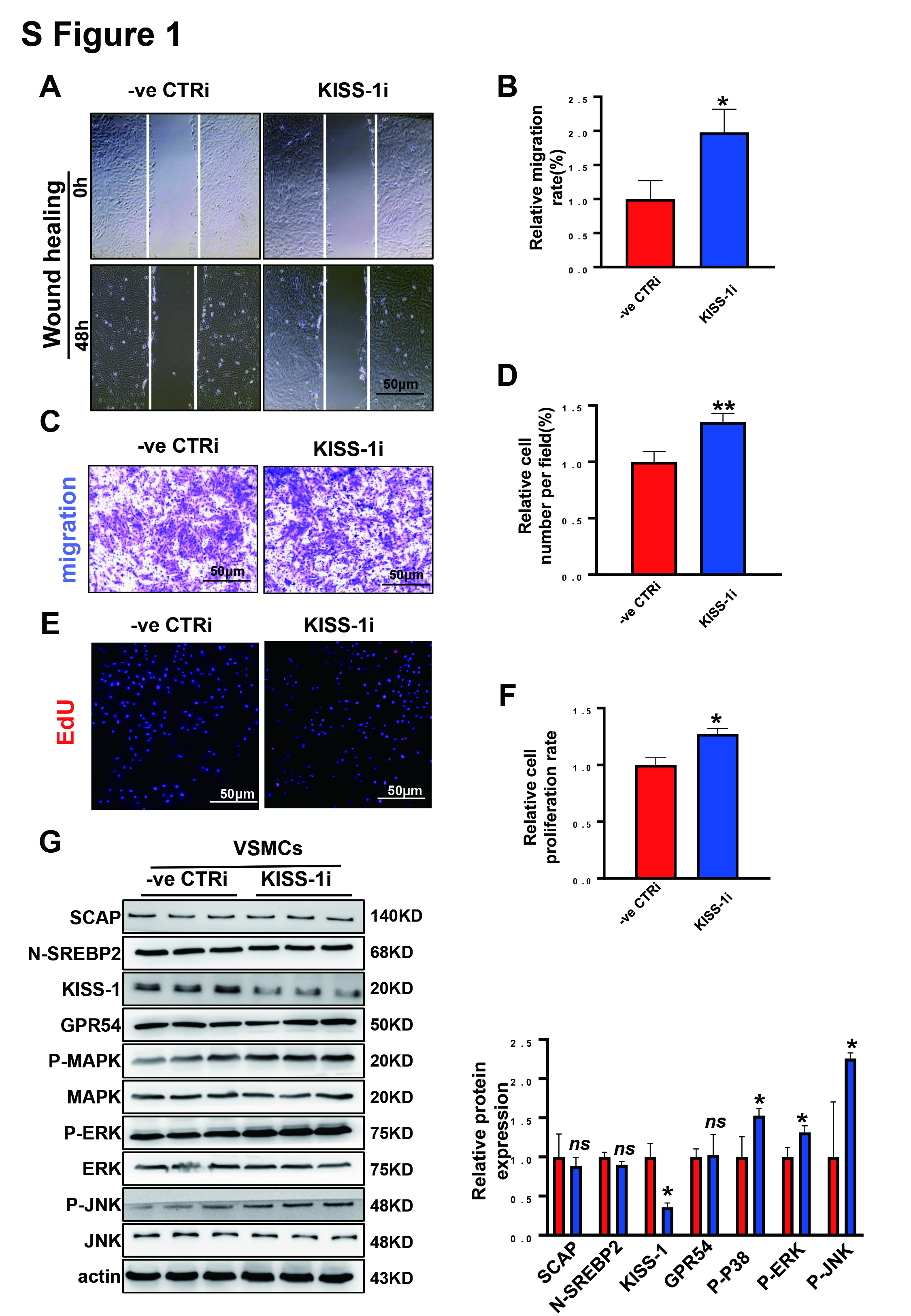

Supplement: Supplementary file 2 — Supplement Figure1 [file 41419_2023_5754_MOESM2_ESM.tif]

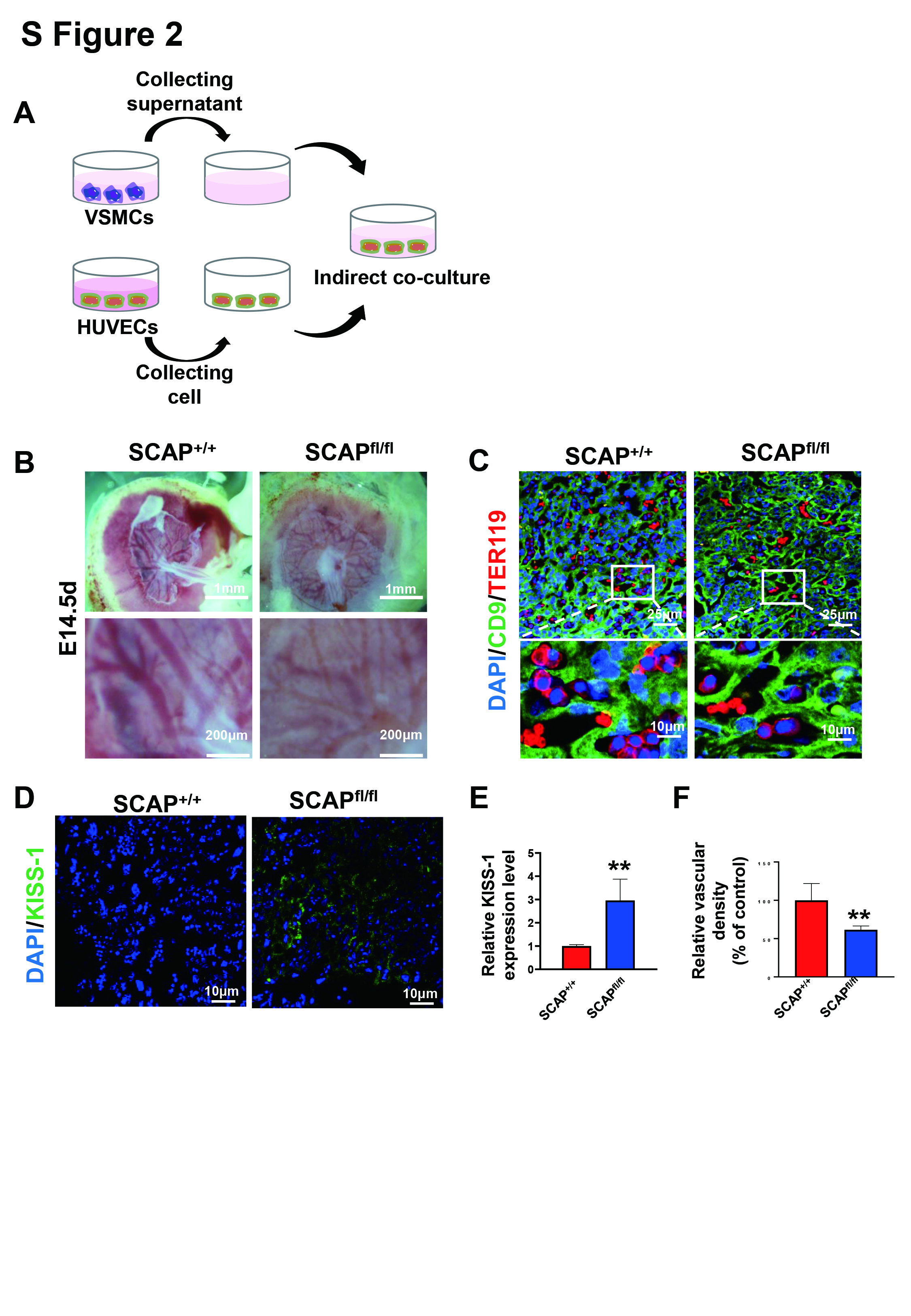

Supplement: Supplementary file 3 — Supplement Figure2 [file 41419_2023_5754_MOESM3_ESM.tif]
