# Supplementary material for: SCAP contributes to embryonic angiogenesis by negatively regulating KISS-1 expression in mice
Source: Cell Death Dis. 2023 Apr 6;14(4):249. doi: 10.1038/s41419-023-05754-8 (PMC10079761; doi:10.1038/s41419-023-05754-8)

Original full length western blots

Figure 3B

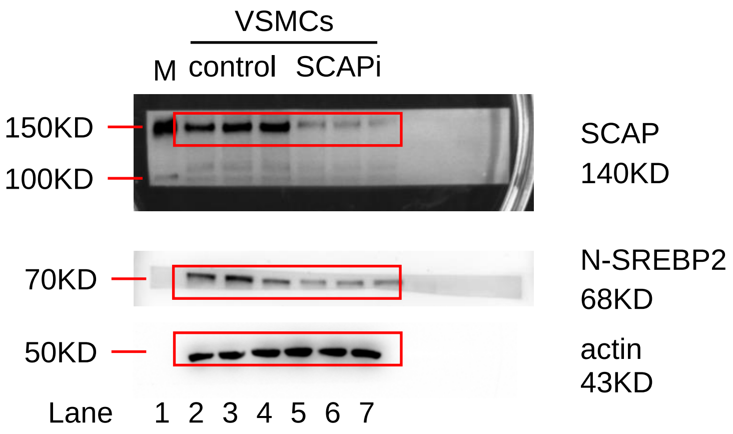

Figure 3L

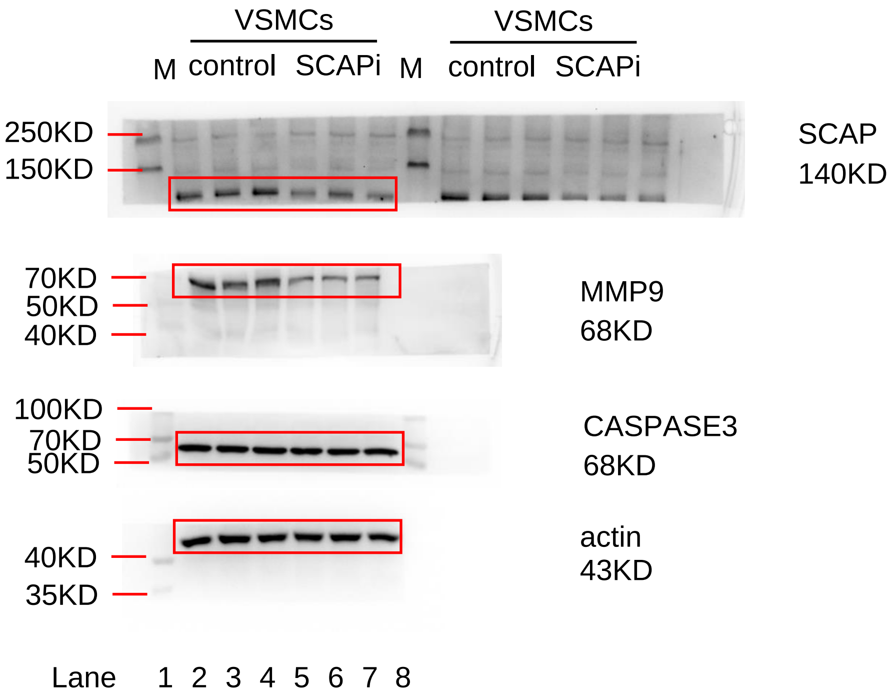

Figure 4E

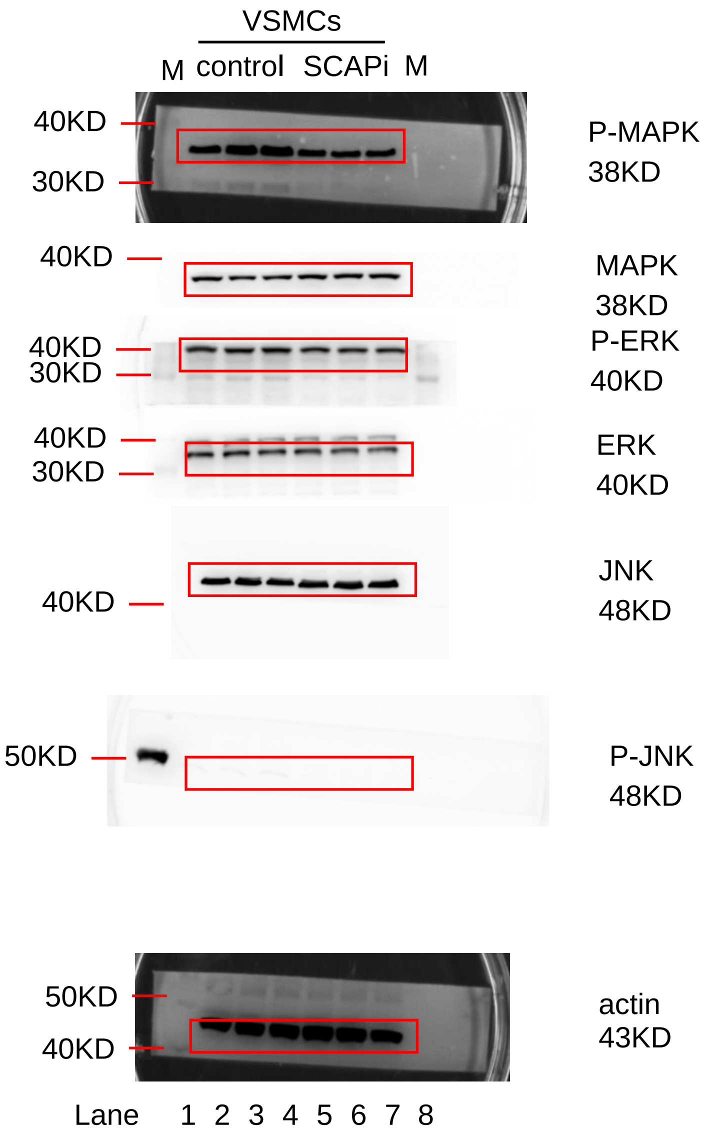

Figure 4G

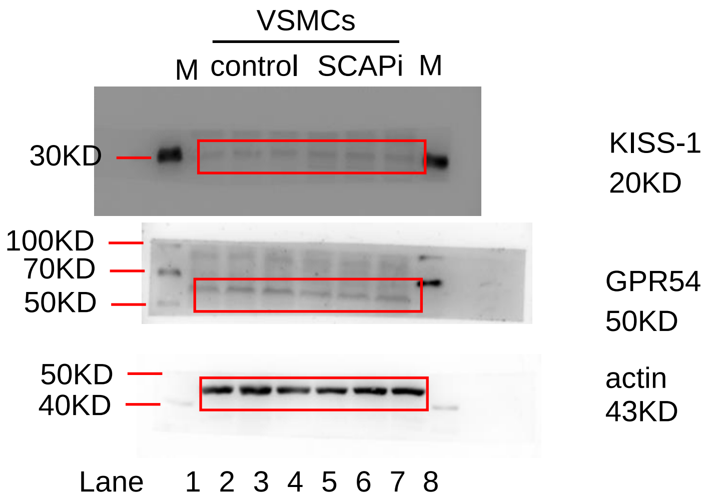

Figure 5G

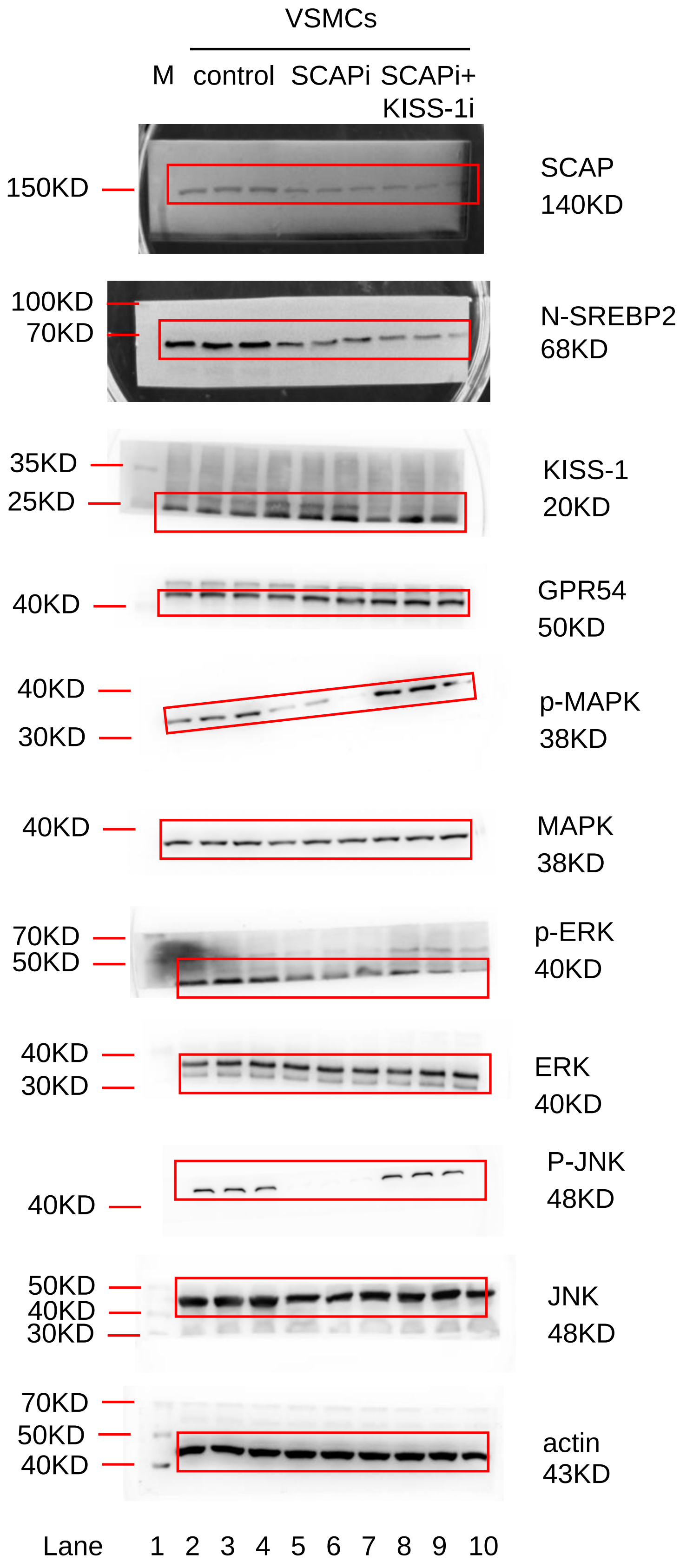
VSMCs
M
control
SCAPi
SCAPi+ KISS-1i
150KD
SCAP 140KD
100KD
70KD
N-SREBP2 68KD
35KD
25KD
KISS-1 20KD
40KD
GPR54 50KD
40KD
30KD
p-MAPK 38KD
40KD
MAPK 38KD
70KD
50KD
p-ERK 40KD
40KD
30KD
ERK 40KD
40KD
P-JNK 48KD
50KD
40KD
30KD
JNK 48KD
70KD
50KD
40KD
actin 43KD
Lane 1 2 3 4 5 6 7 8 9 10

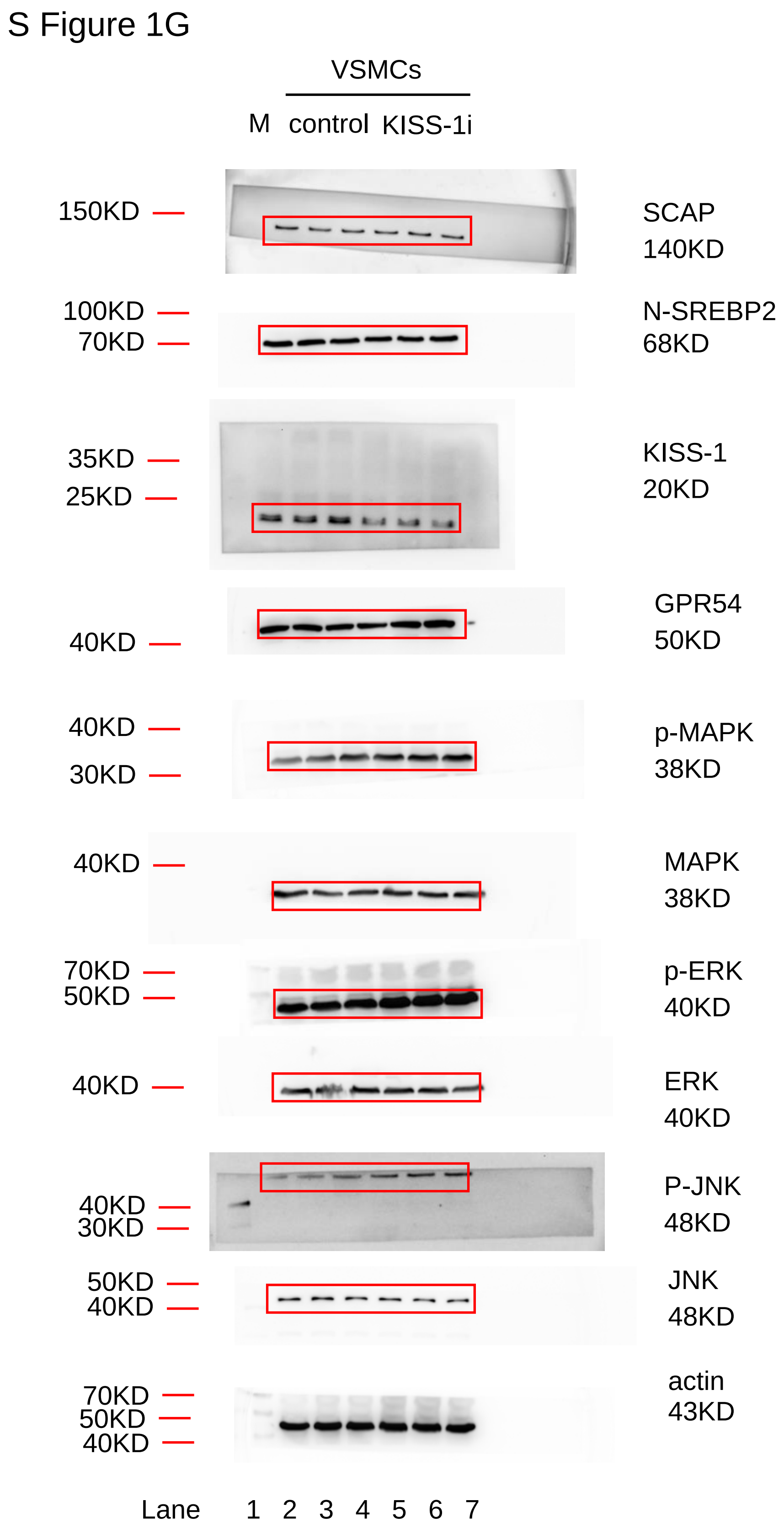
S Figure 1G
VSMCs
M
control
KISS-1i
150KD
SCAP
140KD
100KD
70KD
N-SREBP2
68KD
35KD
25KD
KISS-1
20KD
40KD
GPR54
50KD
40KD
30KD
p-MAPK
38KD
40KD
MAPK
38KD
70KD
50KD
p-ERK
40KD
40KD
ERK
40KD
40KD
30KD
P-JNK
48KD
50KD
40KD
JNK
48KD
70KD
50KD
40KD
actin
43KD
Lane 1 2 3 4 5 6 7

Figure 6D

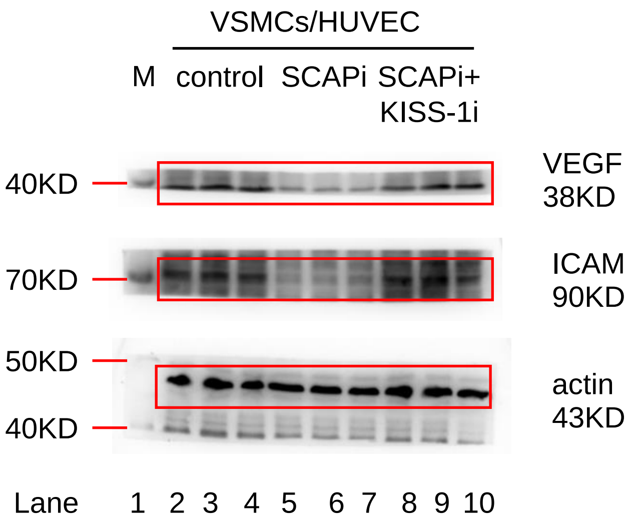
VSMCs/HUVEC
M
control
SCAPi
SCAPi+ KISS-1i
40KD
VEGF 38KD
70KD
ICAM 90KD
50KD
40KD
actin 43KD
Lane
1 2 3 4 5 6 7 8 9 10

Figure 7A

Experiment1

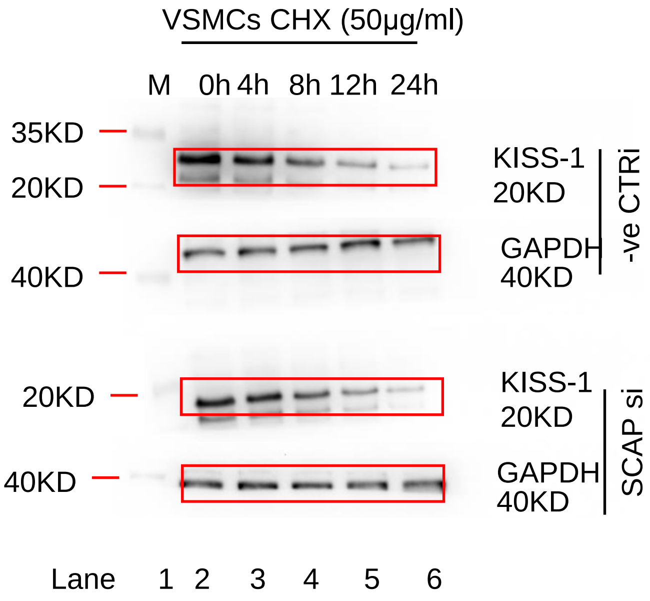

Experiment2

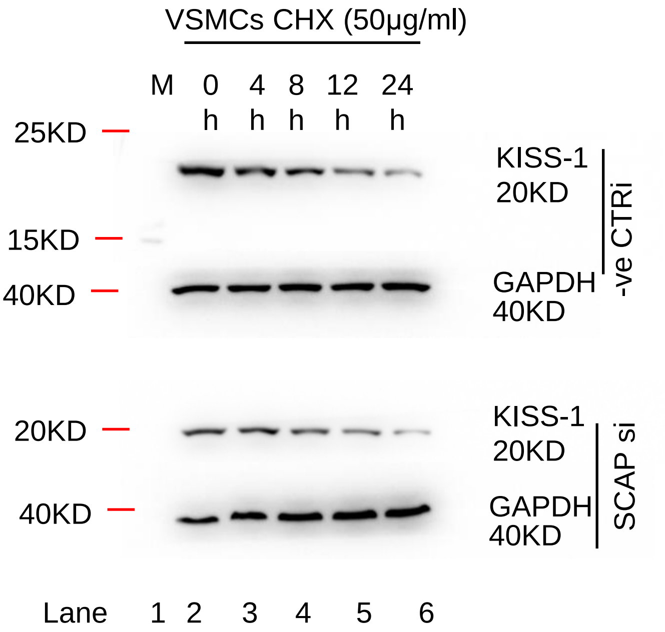

Experiment3

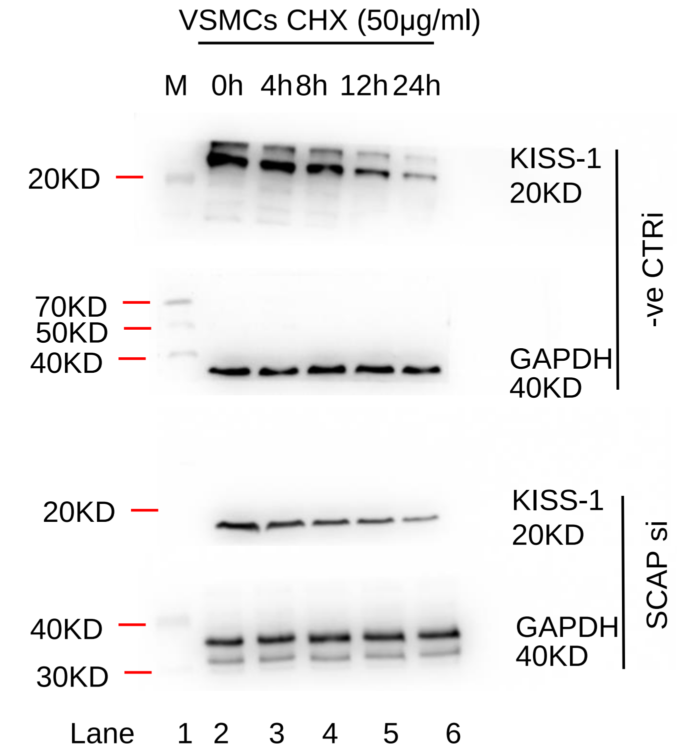

Figure 7D

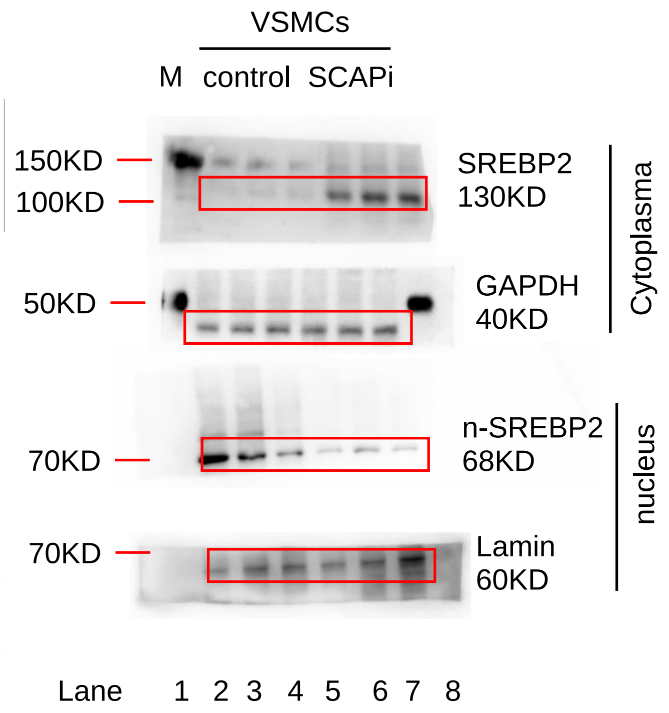

Supplement: Supplementary file 4 — Original western blots [file 41419_2023_5754_MOESM4_ESM.pdf]
